# DDD TinyML: A TinyML-Based Driver Drowsiness Detection Model Using Deep Learning

**DOI:** 10.3390/s23125696

**Published:** 2023-06-18

**Authors:** Norah N. Alajlan, Dina M. Ibrahim

**Affiliations:** 1 Department of Information Technology, College of Computer, Qassim University, Buraydah 51452, Saudi Arabia; 411200285@qu.edu.sa; 2 Department of Computers and Control Engineering, Faculty of Engineering, Tanta University, Tanta 31733, Egypt

**Keywords:** TinyML, deep learning, IoT, driver drowsiness detection

## Abstract

Driver drowsiness is one of the main causes of traffic accidents today. In recent years, driver drowsiness detection has suffered from issues integrating deep learning (DL) with Internet-of-things (IoT) devices due to the limited resources of IoT devices, which pose a challenge to fulfilling DL models that demand large storage and computation. Thus, there are challenges to meeting the requirements of real-time driver drowsiness detection applications that need short latency and lightweight computation. To this end, we applied Tiny Machine Learning (TinyML) to a driver drowsiness detection case study. In this paper, we first present an overview of TinyML. After conducting some preliminary experiments, we proposed five lightweight DL models that can be deployed on a microcontroller. We applied three DL models: SqueezeNet, AlexNet, and CNN. In addition, we adopted two pretrained models (MobileNet-V2 and MobileNet-V3) to find the best model in terms of size and accuracy results. After that, we applied the optimization methods to DL models using quantization. Three quantization methods were applied: quantization-aware training (QAT), full-integer quantization (FIQ), and dynamic range quantization (DRQ). The obtained results in terms of the model size show that the CNN model achieved the smallest size of 0.05 MB using the DRQ method, followed by SqueezeNet, AlexNet MobileNet-V3, and MobileNet-V2, with 0.141 MB, 0.58 MB, 1.16 MB, and 1.55 MB, respectively. The result after applying the optimization method was 0.9964 accuracy using DRQ in the MobileNet-V2 model, which outperformed the other models, followed by the SqueezeNet and AlexNet models, with 0.9951 and 0.9924 accuracies, respectively, using DRQ.

## 1. Introduction

Driver drowsiness is defined as a state of sleepiness when the driver needs to rest, and it can cause symptoms that have a great impact on the performance of tasks, such as intermittent lack of awareness, slowed response time, or microsleeps. While driving, these symptoms are highly dangerous, as they greatly increase the odds of drivers missing exits or road signs, drifting into other lanes, or even crashing their vehicle, causing an accident [1,2,3]. According to the American Automobile Association (AAA), the Foundation for Traffic Safety in the United States reported that driver drowsiness was responsible for 23.5% of all automobile crashes recorded in 2015; 16.5% were fatal crashes, and 7% were non-fatal crashes [4]. Recently, many researchers have proposed systems to be installed in cars to detect driver drowsiness, motivated by the urgent need to limit the number of traffic crashes related to driver drowsiness. Different approaches have been used in research to detect drivers’ drowsiness, including physical features, physiological features, vehicle-based implementations, and hybrid approaches [3]. The physical features approach is the most frequently used to detect drivers’ drowsiness, using features such as eye tracking, yawning, head position, and detecting facial landmarks to detect drowsiness [3].

Many applications integrate DL with IoT devices, including driver drowsiness detection systems in smart vehicles. Driver drowsiness detection is an application that faces challenges once DL is integrated with IoT devices. In particular, detecting drowsiness using videos or images poses several challenges. The authors of [5] performed an extensive survey on driver drowsiness detection systems, and they reported that DL learning models have high computational costs and require a lot of training time. Furthermore, they required a large quantity of data for quality predictions. However, in [6], the researchers recommended the use of lightweight sensors or the analysis of biological signals based on video data to maintain a high accuracy performance.

Several survey and review studies that integrated DL models with IoT devices in driver drowsiness detection systems [5,6,7,8] have reported that the major challenge is training the DL models, in that DL has a complex architecture and is heavyweight. The approximate range of the DL model size in the literature on driver drowsiness detection is from 10 MB to 54 MB. This poses a challenge to deployment on IoT devices that have resource constraints, such as smartphones or Raspberry Pi, which are commonly used in detecting drivers’ drowsiness, as these devices have limited battery energy capacity, small memory size, and limited processor capacity. On the other hand, some studies have used devices that have resource constraints but deployed the DL models to a cloud-based platform, which also poses challenges in terms of latency.

To tackle these challenges, a new and emerging technology called Tiny Machine Learning (TinyML) has paved the way to meet the challenges of integrating DL with IoT devices. TinyML can be defined as an integration of two concepts: machine learning or deep learning and Internet-of-things devices. TinyML technology enables the deployment of DL models on resource-constrained devices powered by microcontrollers. Microcontrollers have low-cost boards equipped with limited computation, extremely low power (mW range and below), and small memory without sacrificing accuracy [9].

To this end, the aim of this research was to apply TinyML to a driver drowsiness detection case in order to overcome the challenge of integrating DL with IoT devices by producing lightweight DL models with a few kilobytes and, thus, enable their deployment on IoT devices that have resource constraints, such as microcontrollers. In this work, we implemented five lightweight DL models; we developed three DL models—namely, SqueezeNet, AlexNet, and CNN models—and adopted two pretrained models (MobileNet-V2 and MobileNet-V3). After that, we executed the optimization methods to reduce the size of the DL models. Two quantization methods were used—namely, post-training quantization (PTQ) and quantization-aware training (QAT). Next, we converted the DL models to TensorFlow Lite (TF Lite) format and used the interpreter to evaluate them.

The rest of this paper is organized as follows: Section 2 presents an overview of TinyML, with mentions of its definition and advantages. Section 3 summarizes the related work on driver drowsiness detection. In Section 4, our research methodology approach is demonstrated. Section 5 presents the implementation and testing of our methodology. Section 6 presents the discussion and comparison of the results. Finally, Section 7 illustrates the conclusions.

## 2. TinyML Overview

Tiny machine learning (TinyML) is an emerging field that culminates in many inventions and leads to the rapid growth of IoT fields, for example, the smart environment, smart transportation, autonomous driving, etc. TinyML is an alternative paradigm that allows deep learning tasks to be implemented locally on ultra-low-power devices that are typically under a milliwatt. Thus, TinyML allows for real-time analyzing and interpretation of data, which translates to massive advantages in terms of latency, privacy, and cost [10,11]. The primary goal of TinyML is to improve the adequacy of deep learning systems by requiring less computational power and fewer data, which facilitates the giant edge artificial intelligence (AI) market and the IoT [11]. According to the universal tech market advisory company, ABI Research [12,13], a total of 2.5 billion devices with a TinyML chipset are expected to be shipped in 2030. These devices focus on advanced automation, low cost, low latency in transmitting data, and ultra-power-efficient AI chipsets. The chipsets are known as intelligent IoT (AIoT) or embedded AI, as they perform AI inference almost fully on the board, whereas in the training phase of these devices, they continue to depend on external resources, such as gateways, on-premises servers, or the cloud.

According to the authors of the book on TinyML [9], TinyML is defined as “machine learning aware architectures, frameworks, techniques, tools, and approaches which are capable of performing on-device analytics for a variety of sensing modalities (vision, audio, speech, motion, chemical, physical, textual, cognitive) at an mW (or below) power range setting while targeting predominately battery-operated embedded edge devices suitable for implementation in the large scale use cases preferable in the IoT or wireless sensor network domain” [14]. A common definition of TinyML is the implementation of a Neural Network model on a Microcontroller or similar devices with a power capacity of less than one mW [15].

TinyML is made up of three main elements (i) hardware; (ii) software; and (iii) algorithms. The hardware can comprise IoT devices with or without hardware accelerators, while these devices can be based on analog computing, in-memory computing, or neuromorphic computing for a better learning experience. Microcontroller units (MCUs) are considered ideal hardware platforms for TinyML due to their specifications [14]. A microcontroller is typically small (∼1 cm), low cost (around 1 USD), and has low power (1 mW) [11,16]. The microcontroller chip combines a CPU, data, program memory (flash memory and RAM), and a series of input/output peripherals. Microcontrollers are used worldwide as most of the desirable characteristics for hardware can be found in these devices. Their MCUs’ clock speed ranges from 8 MHz to about 500 MHz, while RAM ranges from 8 KB to 320 KB, and flash memory ranges from 32 KB to 2 MB. Overall, TinyML uses low-cost devices while it efficiently consumes power and achieves a high level of performance [17]. In terms of Software, TinyML has recently attracted the interest of industry giants. For instance, Google has released the TensorFlow Lite (TFLite) platform, which allows neural network (NN) models to be run on IoT devices [18]. Likewise, Microsoft has released EdgeML [19], whereas ARM [20,21] has published an open-source library for Cortex-M processors that increase the NN performance and is known as the Cortex microcontroller software interface standard neural network (CMSIS-NN). In addition, a new package called X-Cube-AI [21] has been released to execute deep learning models on STM 32-bit microcontrollers [22]. Algorithms such as a deep learning algorithm for a TinyML system should be small (only a few KB). This is by using model compression techniques to reduce the deep learning model size to enable deployment on IoT devices with constrained resources [14].

TinyML achieved successful performance in many fields. It was used for a variety of purposes, including autonomous small cars, traffic management, sign language recognition, handwriting analysis, medical face mask detection, the environment, and more. [14,23].

## 3. Related Work

Several studies have applied various deep learning models, such as CNN, RNN, VGG-16, and AlexNet, to detect driver drowsiness from videos and images captured on IoT devices. For instance, the study in [24] proposed a real-time drowsiness detection system by developing a Sober drive system using an android smartphone. The authors identified the blink rate and eye status, with a back propagation neural network (BPNN) used to classify the eyes status as closed or open. The dataset was created by the authors using five subjects with 60 open-eye images and 60 closed-eye images. The result was 95% detection accuracy in good conditions, but accuracy fell under low illumination and when drivers were wearing glasses.

The authors of [25] used various deep-learning models to detect whether drivers were drowsy or non-drowsy. They applied the public NTH drowsy driver detection (NTHU-DDD) video dataset [13] that contained driving recordings of 36 subjects of different races in five different types of status (bareface, glasses, sunglasses, night_bareface, night_glasses), including normal driving, yawning, slow blinking, and falling asleep [26]. Four deep learning models were utilized, namely, AlexNet, VGG-FaceNet, FlowImageNet, and the long-term recurrent convolutional network (LRCN). The results showed the VGG-FaceNet outperformed the other models with 70.53% accuracy for drivers with glasses. The results for the other models were 70.42%, 68.75%, and 61.61% for drivers with glasses for AlexNet, LRCN, and FlowImageNet, respectively.

On the other hand, the study in [27] proposed a lightweight deep learning model to enable inference in IoT devices to detect driver drowsiness. They created a custom dataset containing 33 subjects for both genders with three different types of statuses for drivers: normal, yawing, and drowsy. The model was 10 megabytes (MB) and was deployed on the Jetson TK1 device, which had 192 computed unified device (CUDA) cores. The experiment achieved 89.5% accuracy and 18.9 milliseconds (ms) of detection time on the Jetson TK1 device. Similarly, the authors of [28] developed a deep learning model which comprised a CNN for inference in IoT devices to detect driver drowsiness. They proposed a model to detect the driver’s status with eyes closed or open. The Closed Eyes in the Wild (CEW) dataset were used, which contained 2,423 subjects, 1,192 with closed eyes and 1,231 with open eyes on the labeled face. They used a Raspberry Pi3 device to establish a region of interest in the face and to detect the eyes in real-time, with an alert sent later to the android phone. The results indicated 95% accuracy. In addition, the authors of [29] proposed a system to recognize three types of driver status: distraction, fatigue, and drowsiness. They proposed MT-Mobilenets, an improved method from the Mobilenets used in previous research. The MT-Mobilenets model depended on facial recognition using two cases, namely, drowsiness and distraction, without the need for face detection and recognized facial behaviors independently as mouth opening, eyes closure, and head position. The authors created their dataset using a driving simulator with 12 subjects ranging in age from 20–50 years. The total dataset comprises 38,945 images divided into 20,000 images for training and 18,954 images for testing. They used a Raspberry Pi device that had a four-core Cortex A53 CPU and did not have a graphics processing unit (GPU). The accuracy of MT-Mobilenets was 94.44% for drowsiness, 98.96% for distraction, and 84.89% for fatigue.

The other problem to solve was a limitation of the intensive computation required for the integration of deep learning models with IoT devices. The authors of [30] proposed a real-time lightweight deep learning model to detect driver drowsiness using a smart phone. They used the multi-task cascaded convolutional neural networks (MTCNN) technique to locate the driver’s face, eyes, mouth, and nose from input images, with these then fed to a lightweight CNN model to detect if the driver was drowsy or non-drowsy. The dataset created by the authors in the simulated driving set-up consisted of 62 subjects and contained 145k images. The experiment first ran the MTCNN on ARM-NEON, then ran the proposed CNN on Mali GPU on a smartphone to detect driver drowsiness. The size of the proposed model was 19.16 MB: overall, this model achieved 94.4% accuracy and real-time performance of 60 frames-per-second with computational 650× on the smartphone. The author of [31] used a lightweight deep-learning model to detect driver drowsiness by utilizing facial landmarks. Lightweight VGG-16 and Alexnet models were used to classify the drowsiness status on smartphones (Galaxy-8) and embedded devices. They used the NTHU dataset, which contained various types of driver drowsiness statuses, such as talking, yawning, slow-rate blinking, sleepy head movements, and closed eyes. Various conditions were applied, such as recording drivers during the day and at night. After performing pre-processing on the videos and extracting the images, the model size was 236 MB and 547 MB for the VGG-16 and Alexnet models, respectively. The overall classification showed 83.33% accuracy. Moreover, the accuracy performance was 85.82%, 88.89%, 83.76%, 79.45%, and 78.72% for the categories of the night without glasses, without glasses, with glasses, night with glasses, and with sunglasses, respectively. Furthermore, the study in [32] proposed a deep learning model to detect driver drowsiness using a smartphone. The authors proposed depthwise separable three-dimensional (3D) convolutions, combined with an early fusion of spatial and temporal information, to achieve a balance between high accuracy performance and real-time inference requirements. The authors used the academic NTHU dataset [13], which comprised five types of driver status: namely, a driver without glasses, with glasses, with sunglasses, without glasses at night, and with glasses at night. In addition, the dataset contains simulated behaviors such as yawning, looking aside, nodding, laughing, talking, closing eyes, and normal driving. The dataset had 18 subjects for training, four subjects for evaluation, and 14 subjects for testing sets. The model was deployed on the Samsung Galaxy S7 smartphone. The average accuracy for all types of driver status was 73.9%. The accuracy for the other type of driver status was 75.4%, 77.4%, 76.8%, 76.1%, and 63.6% for without glasses, with glasses, with sunglasses, without glasses at night, and with glasses at night, respectively.

The study in [33] presented a driver drowsiness detection framework that placed the vehicle as a standalone design unit. The framework comprised two distinct phases. The first phase was image data acquisition, followed by the identification of the region of interest (ROI) phase using IoT devices. The second phase used a deep learning model to classify and predict the outcome. In the first phase, the hardware for acquiring the images comprised a Raspberry Pi4 device, a Pi camera module v1.3, with a buzzer: the Haar feature-based cascade classifier OpenCV algorithm was used for detection. The images were then fed to the second phase, which used a CNN model to predict whether the driver was or was not drowsy. The dataset contained 7000 images from various open-source eye image datasets, including drowsiness detection and eye state detection datasets. The results depicted 95% to 96% of accuracy. The authors of [34] developed deep learning models to detect driver drowsiness through extracts of the mouth region yawing or non-yawning in real-time. Firstly, they used Dlib’s frontal face detector and a custom Dlib landmark detector to extract the mouth region from a video stream. Secondly, they extracted deep learning features using a deep convolutional neural network (DCNN). Lastly, they used a yawn detector composed of 1D-depthwise separable-CNN and a recurrent gated unit (GRU) to predict if the driver’s status was yawning or not yawning. Three datasets were used, namely, AffectNet, the yawning detection dataset (YawDD), and iBUG-300 W. The experiment was first conducted on a host computer and Latte Panda with an Ubuntu-embedded device. It was then tested using a host computer and an embedded device with a live video feed, the YawDD, and the NTHU dataset. The performance result of the yawning detector achieved 99.97% accuracy. However, the results of inference models on the host computer and embedded board were 30 and 23 frames per second (fps), respectively.

Previous studies that focused on determining eyelid and mouth movements reported their limitations. This included the limitations of physiological measures that may not be workable in practice, as the measuring devices were not comfortable for drivers and often were not available in vehicles. The authors of [35] proposed two adaptive deep neural networks, namely, drowsiness or non-drowsiness, to detect the status of drivers in real-time. Firstly, they performed pre-processing by detecting faces using a single-shot multibox detector (SSD) network with the ResNet-10 technique. Secondly, they detected driver status using two adaptive deep neural networks, namely, MobileNet-V2 and ResNet-50V2 models. The authors created a dataset comprising images and videos of drowsy and non-drowsy faces recorded by cameras in Kaggle, Bing Image Search API, iStock, and the real-world masked face dataset (RMFD). The dataset contained 6,448 images in a ratio of 80% (5158) for training and 20% (1290) for testing. The experiment was conducted on an Nvidia Geforce GPU with 8 gigabytes (GB) of remote access memory (RAM). The experiment showed the ResNet-50V2 model outperformed the other model with 97% accuracy and a running time of 5.1 seconds (s), whereas the MobileNet-V2 model had 96% accuracy with a low running time of 4.3 seconds (s). 

The detection performance of driver drowsiness methods decreased once complications occurred, for example, variations in the driver’s head pose, illumination changes inside the vehicle, occlusions, or shadows on the driver’s face. Previous methods did not have the capability to distinguish between the driver’s status, such as blinking versus closing eyes or talking versus yawning. The authors of [36] proposed a novel and robust framework for driver drowsiness detection using a two-stream spatial–temporal graph convolutional network (2s-STGCN). The framework comprised two stages, the first stage being the detection of the driver’s facial landmarks from a real video. In the second step, the driver’s consecutive facial landmarks were fed to the trained 2s-STGCN model. Two datasets were used: namely, the yawning detection dataset (YawDD) and the NTHU-DDD dataset. The average accuracy was 93.4% on YawDD, while the average accuracy was 92.7% on the NTHU-DDD dataset.

The recent research documented in [37] implemented the deep learning model on an IoT device, namely, a Raspberry Pi device, to classify drowsiness symptoms of drivers, that is, blinking and yawning. They proposed a CNN model consisting of a 4 -layer convolution. The dataset contained 1,310 images that were used to train the CNN model. The real-time experiment was conducted on 10 subjects to obtain the effectiveness of the proposed model. The CNN model successfully demonstrated a classification accuracy rate between 80% and 98%. The study in [38] proposed a system for driver drowsiness detection based on integrating deep learning frameworks with IoT devices. The system comprised three phases: eye region detection, eye status detection, and classification. If the driver was drowsy, the alert system was used to notify him/her. The study first used a faster region-based convolutional neural network (f-RCNN) for detection, if the background was complicated, of the eye region in facial images of the driver. After that, only the eye region was fed into a CNN model to detect if the eye status of the driver showed drowsiness or non-drowsiness. Lastly, an alert system using an Atmega328p microcontroller was generated based on the drowsiness level of the driver’s eye status. The authors created their dataset using 24 subjects comprising both males and females. The proposed model’s performance achieved 97.6% accuracy.

Furthermore, the study in [39] developed a driver drowsiness detection system using a Raspberry Pi device, which detected and counted the driver’s mouth opening, eye blinking, and closing to detect drowsiness. When the driver closed his/her eyes for an extended time, an alert sound was generated to notify him/her. Moreover, the vehicle’s owner was notified by email if the driver was observed to be dozing off more than a few times. The authors used Dlib based on a CNN to detect drowsiness. The overall real-time performance of the system achieved 96% accuracy.

In the study presented in [40], a CNN deep model was developed that had 14 layers with 1,236,217 parameters to detect driver drowsiness using mobile devices. The authors developed their model on mobile devices due to their widespread use and low power consumption. They detected driver drowsiness through the driver’s eyes: if the driver’s eyes were closed for more than three seconds, the driver was warned with a message and an alarm sound. The Closed Eyes in the Wild (CEW) dataset was used, which contained 2425 images in two categories comprising open-eye and closed-eye images. The model developed for drivers’ drowsiness detection obtained 95.65% accuracy.

Based on the previous studies in aforementioned above, we conclude that a number of studies used heavy-weight deep learning models ranging from 10 MB to 54 MB. Most of the devices used to deploy deep learning models are smartphones and Raspberry Pi, which have high resources. The Raspberry Pi device specifications are 8 GB of Flash memory, 4 GB of RAM, and GPU with 2.4 GHz, and power consumption with 3A and above. While for Smart Phone devices, the specifications are 64 GB, 4 GB of RAM, GPU with 2.3 + 1.7 GHz, and a power consumption of 3000 mAh. However, some studies have used the embedded device or microcontroller as a sensor for the data collector. After that, the microcontroller sends the data to the cloud-based platform for the processing phase by using deep learning models.

## 4. Methodology

**Figure 1:**
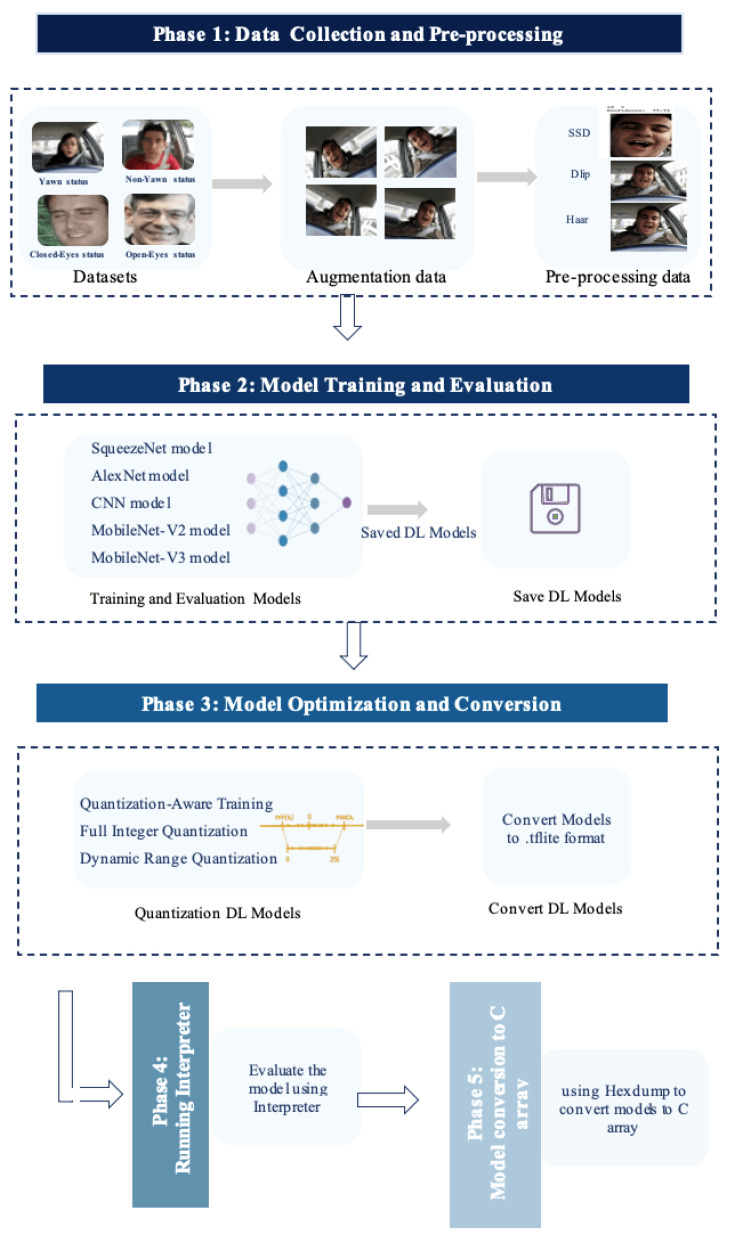
Proposed DDD TinyML Methodology.

This section describes the proposed methodology of the study’s experiment on the driver drowsiness detection case using the TinyML (DDD TinyML) model. The aim is to detect driver drowsiness using small deep-learning models. The methodology’s workflow has several phases, with each phase linked to the next phase. Figure 1 illustrates the implementation phases of the proposed method, while details of the implementation are described below:

- Phase 1: Data Collection and Pre-processing:

In this phase, two datasets are used to detect the status of the mouth and eyes of the driver, indicating if the driver is yawning or non-yawning and with closed or open eyes, as shown in Figure 1. Subsequently, using augmentation techniques, the sample size of images in the datasets is increased. Then, pre-processing of the datasets is implemented by detecting the faces using three methods, namely, single shot multibox detection (SSD), the Dlip library, and the Haar feature-based cascading classifier for these images to be used as input for deep learning models to detect the driver’s status.

- Phase 2: Model Training and Evaluation:

In this phase, three supervised deep learning models are developed to detect the driver’s status, aiming to examine the model’s performance and obtain the best model in terms of size and accuracy. The three deep learning models developed are SqueezeNet, AlexNet, and CNN; furthermore, the study adapts two pre-trained models, namely, MobileNet-V2 and MobileNet-V3, with their pre-training conducted on the ImageNet dataset. Subsequently, all models are saved to be fed to the model optimization phase.

- Phase 3: Model Optimization and Conversion:

This phase aims to optimize the saved deep learning models by reducing the size of models, after which they are converted to Tensor Flow Lite (TFLite) format. Firstly, the saved models are optimized by using several quantization methods, namely, dynamic range quantization (DRQ), full integer quantization (FIQ), and quantization-ware training (QAT), as described in the next section. Quantization aims to convert the weights of models or activation or both from 32-bit floating-point numbers to 8-bit integer format, which provides a significant reduction in model size, thus leading to a decrease in the device’s memory footprint in devices. Secondly, the deep learning models are converted to TFLite format (.tflite) to enable inference of these models.

- Phase 4: Running TFLite Interpreter:

In this phase, the TensorFlow Lite (TFLite) Interpreter is run to evaluate the TFLite deep learning models on the host computer. This is conducted by loading the TFLite model to the Interpreter and using the testing dataset for evaluation.

- Phase 5: Models’ Conversion to C Array:

In this phase, the TFLite models are converted using TensorFlow Lite (TFLite) Micro tools that convert deep learning models to C byte array. This is conducted by using XXD to generate a C source file for the TFLite models as a char array. The models are then deployed into an independent platform using C++ language in Arduino software and compiled with IoT devices as microcontrollers.

## 5. Implementation and Testing

### 5.1. Implementation Environment

The environment in which the experiment was performed was Google Colaboratory (Colab) [41,42], a product of Google Research. Colab allows one to write and execute arbitrary Python code through the browser. Furthermore, Colab allows access to files stored in a Google Drive account. Google produced the Colab pro version [43], which was chosen for the execution of the experiment in this paper due to the features of the environment provided. Colab pro version came with 2 terabytes of storage, 25 gigabytes of random-access memory (RAM), and a GPU (graphics processing unit) processor P100. The experiment was performed using open-source libraries and artificial intelligence (AI) frameworks. In addition, the experiment was written in Python3 programming language and used NumPy [42], Matplotlib [44], Panada [45], and Scikit-learn [46] libraries. The frameworks used were TensorFlow [47], TensorFlow Lite [48], and TensorFlow Lite Micro [49] frameworks, which are described in the next section.

### 5.2. Experiment and Implementation

The following subsections explain in detail the implementation of each phase for the proposed methodology: Phase 1 (data collection and pre-processing); Phase 2 (model training and evaluation); Phase 3 (optimization and conversion model); Phase 4 (running the interpreter); and, finally, Phase 5 (convert of the model to C array).

#### 5.2.1. Phase 1: Data Collection and Pre-Processing

The phase of data collection and pre-processing of the datasets aims to collect a good training dataset for the driver drowsiness detection case. In addition, the paper performs pre-processing on drowsiness and non-drowsiness images in the datasets. This optimizes the achievement of a higher level of performance in the training and evaluation of deep learning models. This phase introduces the detail of the collected datasets and then presents the steps for datasets Pre-processing.

- The collected dataset

The driver’s status ranges between drowsiness and non-drowsiness, with the drowsiness status showing signs such as yawning or closed eyes. In contrast, the signs for a driver in the non-drowsiness or awake status drivers are non-yawning or open eyes. In this research, two datasets are accessed and collated for the experiment to detect the driver’s status. The first dataset is the yawning detection dataset (YawDD) [50], which provides videos of drivers sitting in the driver’s seat with different mouth expressions, such as normal, talking, and yawning. The videos are taken in real conditions with varied illumination. The dataset contains videos of 107 participants: 57 males and 50 females of different ages and ethnicities and with different facial characteristics. Furthermore, videos are taken of participants wearing prescription eyeglasses, wearing sunglasses, and without glasses. The current study uses a dataset from Kaggle [51], which converts the YawDD videos into images. The dataset includes two categories of driver status, namely, yawn and non-yawn, with the non-yawn status including normal status and talking status. The second dataset is the Closed Eyes in the Wild (CEW) dataset [52], which contains 2423 male and female subjects with closed and opened eyes, as well as some wearing prescription glasses and some without glasses.

The collected dataset outcomes for each status of drivers are 726 images of yawn and 726 images of non-yawn, whereas 1192 images are of closed eyes and 1231 images are of open eyes. The total dataset has 3875 images, 70% for the training dataset and 30% for the testing dataset. Table 1 illustrates the number of images per driver status and the size of the datasets.

Moreover, the study employs geometric data augmentation techniques to increase the sample of images to overcome the limitations of the samples available. In addition to improving the deep learning models’ performance, augmentation techniques are used to rescale the images and adjust the zoom range to 20 degrees, as well as provide a True value for a horizontal flip. Table 1 depicts the number of augmentation datasets for each driver status.

- The pre-processing of the dataset

**Figure 2:**
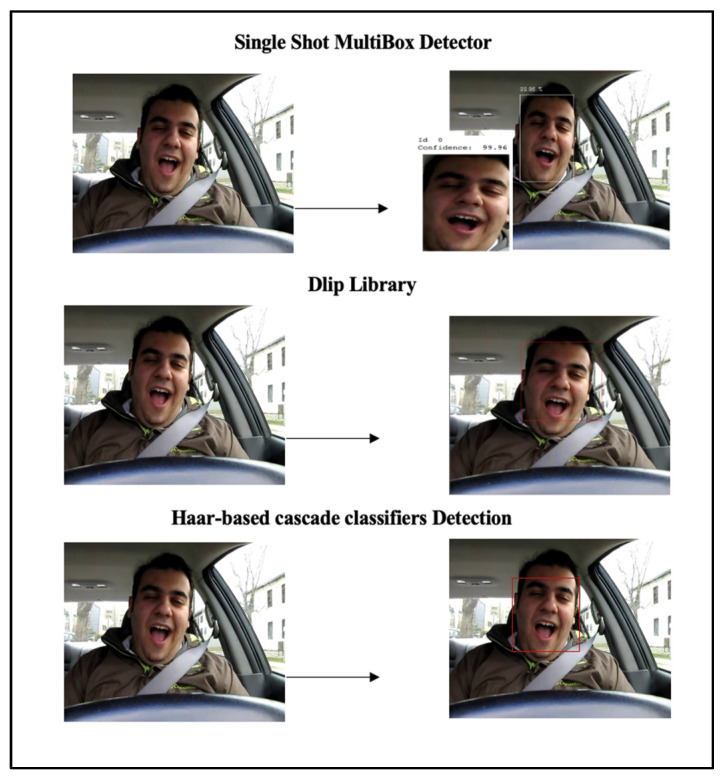
Pre-processing methods: examples from datasets: single shot multibox detector (SSD), Dlip library, and Haar feature-based cascade classifiers.

Pre-processing is widely used to prepare datasets and create good training datasets so the achievement of high performance can be promoted in the training and evaluation of deep learning models. The current study performs pre-processing on four categories of driver drowsiness detection images: yawning, non-yawn, closed eyes, and open eyes. The preprocessing passes through several steps. Firstly, it uses various face detection methods to detect drivers’ faces in the dataset, namely, single shot multibox detector (SSD), Dlip library, and Haar feature-based cascade classifiers detection in OpenCV as shown in Figure 2, with the details of each described as follows:

- Single Shot MultiBox Detector (SSD) Network [53]: A more accurate method for detecting drivers’ faces in images is to use the single shot multibox detector (SSD) network by assigning the model file to pre-train a ResNet-10 model with a config file. The dataset is then set as input for the model so it can detect the face. The SSD method is based on a feed-forward convolutional network that produces a fixed-size collection of bounding boxes and scores for the presence of object class instances in those boxes, followed by a non-maximum suppression step to produce the final detections.
- Dlip Library [54]: The Dlip Library is used to detect the face area from images. Dlip is a platform-independent programming library based on C++ with a Python interface [54]. The current study uses features of a pre-trained histogram of oriented gradients (HOG) and a linear classifier support vector machine (SVM) face detector from the Dlip library. For detection of the driver’s face from images, a frontal face detector is created through which the dataset image passes.
- Haar-based cascade classifiers Detection [55]: The Haar feature-based cascade classifiers in the OpenCV method were proposed by Viola and Jones to detect the object [56]. In this machine learning-based approach, a cascade function is trained from many positive images (images with faces) and negative images (images without faces). These are used to detect objects in other images. The current study uses pre-trained Haar cascade models to detect drivers’ face images in the dataset [57] by loading the Haar cascade file of the frontal face using the CascadeClassifier method of the CV2 module.

The detection of face techniques process resizing all the images, followed by reading and resizing images of the Eyes dataset. The two datasets are then combined. The second step reshapes images so they are appropriate as inputs to deep learning models. The third step uses the LabelBinarizer method to transform multi-class labels into binary labels, which redefines the category label in datasets to binary. In the fourth step, the dataset is randomly split into 70% and 30% for training and testing, respectively, to insure the diversity of images. In addition, the seed for the random generator is set at 40 to ensure the results can be reproduced. Finally, the augmentation method is used for the training dataset by rescaling the images and adjusting the zoom range to 20 degrees. Similarly, the rotation range for images is equal to 30 degrees, as well as providing a True value for the horizontal flip. This is followed by providing a True value for shuffling the data. In addition, the batch size, which refers to the number of training examples utilized in one iteration, is equal to 32. Furthermore, the testing dataset uses MinMaxScalar class for scaling images.

#### 5.2.2. Phase 2: Model Training and Evaluation

Several types of supervised deep learning models are developed in the current to detect driver status, namely, yawn, non-yawn, closed eyes, and open eyes, aiming to obtain the best performance. The current study develops five small, lightweight deep models while preserving their performance. The deployment devices experience size constraints after performing a large amount of experimentation on the architecture of several deep models and comparing their performance. The three deep models are developed, namely, SqueezeNet, AlexNet, and CNN, with the study adapting two pre-trained models, namely, MobileNet-v2 and MobileNet-v3, that are pre-trained on the ImageNet dataset. The following subsections describe the architecture of the deep models used in the experiment.

##### SqueezeNet Deep Model

**Figure 3:**
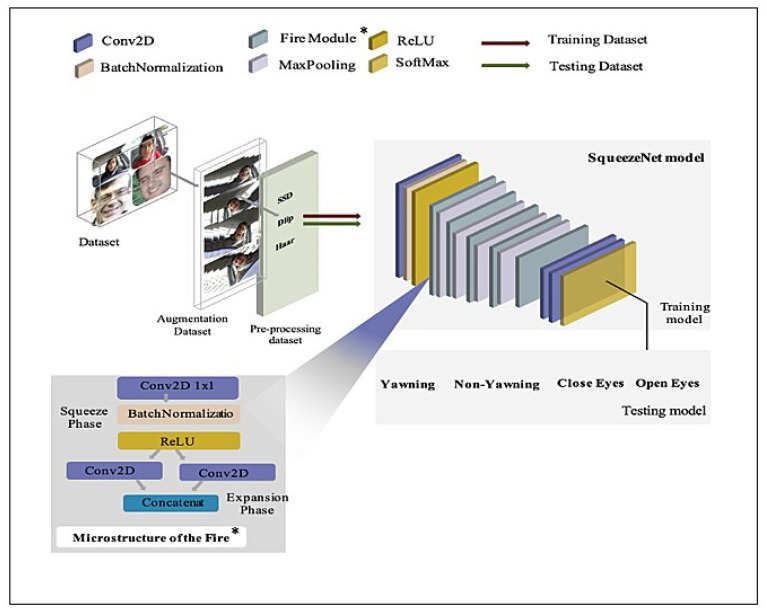
SqueezeNet deep model architecture.

The SqueezeNet deep model is selected in this study as it is a lightweight, efficient model. The main aim of the SqueezeNet deep model is to produce architecture with few parameters while maintaining competitive accuracy, hence, producing a lightweight deep learning model [58,59]. The current study modifies the original SqueezeNet model architecture so it is lighter than the original model while maintaining accuracy to be able to detect driver drowsiness. The authors in [59] have called the Fire Module nine times, while in our research, it is called only five times. Also, a modification has been done by deleting the batch normalization and adding expanded layers to the activation function. The basic idea of SqueezeNet model architecture is a Fire Module that comprises a squeeze convolution layer with only a (1 × 1) filter, feeding to an expanded layer that has a mix of (1 × 1) and (3 × 3) convolution filters, as shown in Figure 3. The ReLU activation function is included with each layer. The SqueezeNet model architecture first comprises a stand-alone convolution layer with input shape (192 × 192 × 3), followed by a BatchNormalization layer and the ReLU activation function. MaxPooling layers with strides equal to two values come after each of the five Fire Modules. The model is completed with a GlobalAveragePooling2D layer and a Dense layer with SoftMax activation function to detect driver drowsiness status, as illustrated in Figure 3. Table 2 illustrates the SqueezeNet model architecture and the number of parameters. The parameters total 118,900, which are split into 118,420 trainable parameters and 480 non-trainable parameters.

##### AlexNet Deep Model

**Figure 4:**
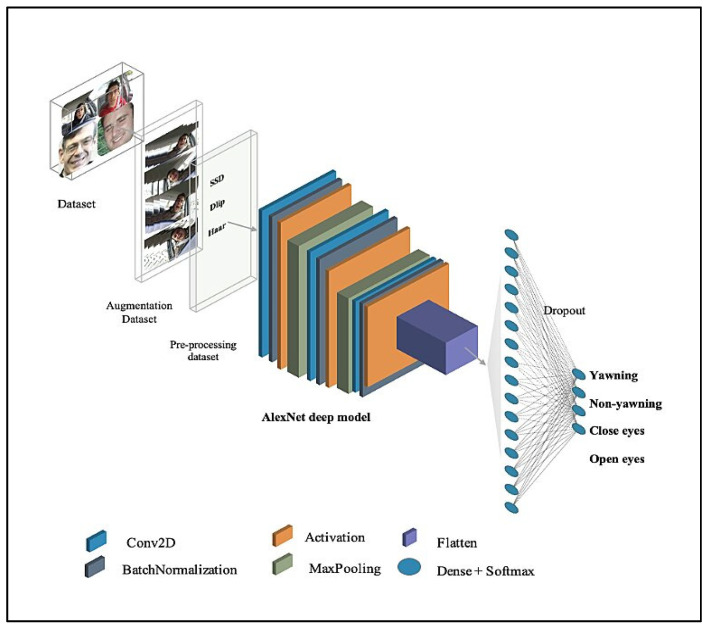
AlexNet deep model architecture.

The AlexNet model is one of the lightweight models which have few parameters. It is selected due to its high level of performance, even with its small size, in previous TinyML studies. It is also used in driver drowsiness detection research, where it has achieved good performance. The current study modifies the AlexNet model architecture so it is smaller in size and has few parameters [60]. The architecture comprises six layers with an input shape of (192 × 192 × 3). The first three layers are convolution layers, each layer followed by a BatchNormalization layer, a ReLU activation layer, and MaxPooling layers with a pool size equal to two, strides equal to two values, and padding that has a valid value. These are followed by a Flatten layer and a Dense Layer, with Dropout having a 0.4 value. The final layer is an output layer which is a Dense layer containing a SoftMax activation function to detect driver drowsiness status, namely, yawn, non-yawn, closed eyes, and open eyes. Table 3 demonstrates the AlexNet model architecture and the number of parameters. The parameters total 598,980, which are split into 598,532 trainable parameters and 448 non-trainable parameters. Figure 4 presents the architecture of the AlexNet deep model.

##### CNN Deep Model

**Figure 5:**
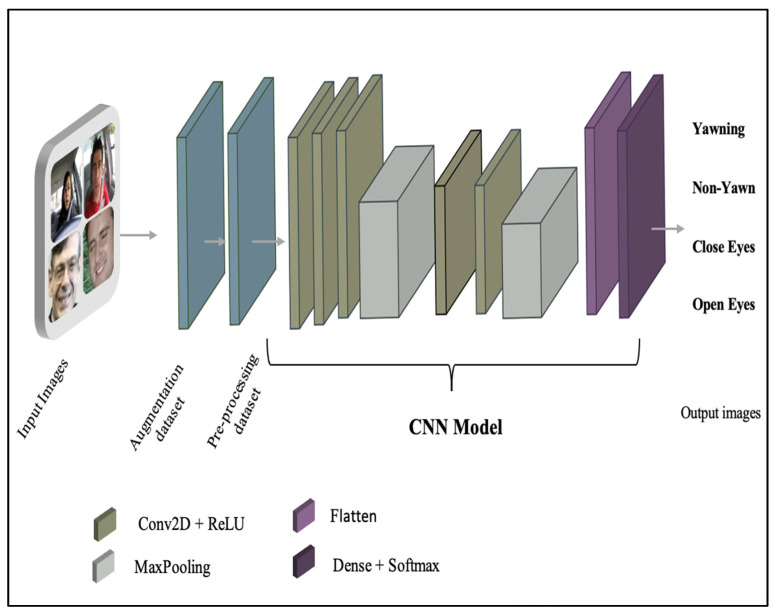
CNN deep model architecture.

The convolution neural network (CNN) deep model is designed to classify and analyze visual data. Overall, it has shown effective results with pattern identification and the problem of feature extraction [61]. In the current study, the CNN deep model is proposed for detecting driver drowsiness status while maintaining model size with performance. The CNN model architecture comprises of input layer that receives input images in four categories (145 × 145 ×3). Three 2D convolutional layers consist of the learned kernels (weights), the extracted features that differentiate different images from one another, and ReLU, the activation function. In addition, two layers of the MaxPooling type are added between 2D convolutional layers, with the pool size set to 2 and padding having a valid value. The outputs of the convolutional layers are passed to flatten the layer, transforming the shape of data into a one-dimensional (1D) data vector. The final layer is an output layer, namely, a Dense layer that contains the SoftMax activation function for detecting driver drowsiness states, namely, yawn, non-yawn, closed eyes, and open eyes). The proposed architecture of the CNN model can be seen in Figure 5. The parameters in the CNN model total 46,574, with all parameters being trainable parameters with zero non-trainable parameters. Trainable parameters are the parameters which are updated during the training process to obtain optimal values. However, non-trainable parameters are not updated and optimized during the training process: as a result, they do not contribute to the classification process. Table 4 shows the CNN model architecture and the number of parameters.

##### MobileNet-V2 Deep Model

**Figure 6:**
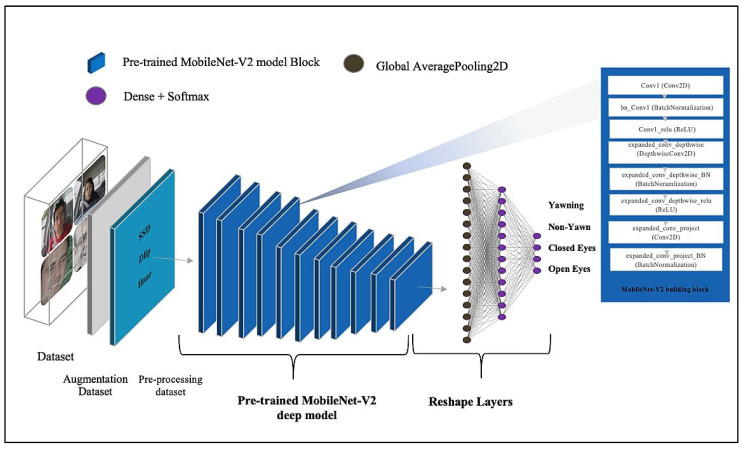
MobileNet-V2 deep model architecture.

The MobileNet-V2 deep model [62] is an improved version of the MobileNet-V1 model, which enhancement of the image classification and recognition capabilities on mobile devices. The MobileNet-V2 model is selected due to its advantages. It is fast, lightweight, and has high accuracy; hence, it is appropriate for training with limited datasets. This model is pre-trained on datasets such as ImageNet. The current study uses the pre-trained MobileNetV2 model on the ImageNet dataset, with the initial weights helping to attain a faster training time and higher accuracy. The current study uses a pre-trained model with weights and re-trains it on the training dataset to fine-tune its parameters. It thus meets the requirements of a large dataset and leads to faster learning. The model architecture consists of a pre-trained MobileNet-V2 model with input shape (224 × 224 ×3) and an alpha value of 0.75. The trainable parameters of the model are False. This is followed by the reshaping layers, which are the MaxPooling 2D layer, Flatten layer, and Dense layer with SoftMax activation function to detect the driver drowsiness status. Table 5 and Figure 6 both contain more information regarding the architecture of the model. The parameters total 1,428,148, which are divided into 46,084 and 1,382,064 for trainable parameters and non-trainable parameters, respectively.

##### MobileNet-V3 Deep Model

**Figure 7:**
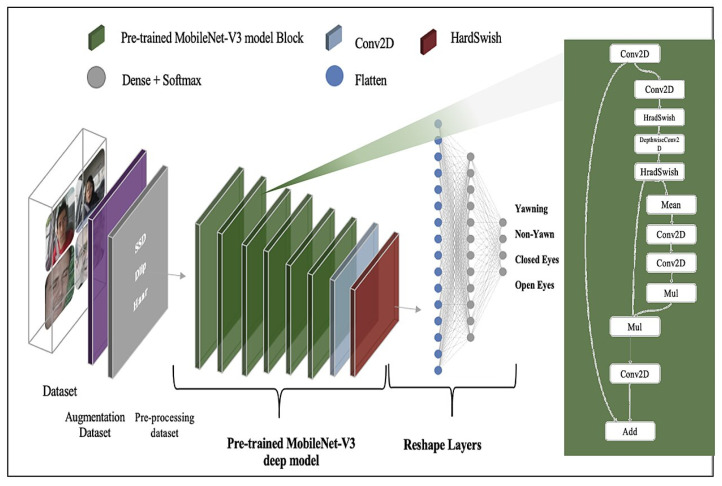
MobileNet-V3 deep model architecture.

The MobileNet-V3 deep model [63], originally published by Google, belongs to the family of neural network architecture, especially for efficient on-device image classification and related tasks. MobileNet-V3 is an updated version of MobileNet-V2. MobileNet-V3 is selected in the current study as the model’s aim is to develop mobile computer vision architecture to improve accuracy latency on mobile devices. Google produced two versions of MobileNet-V3, namely, MobileNet-V2 large and MobileNet-V3 small. The current study uses the pre-trained MobileNet-V3 small model from the Keras library. The adjusted model consists of pre-trained weights on the ImageNet dataset, an alpha value equal to 1.0, and includes a top equal to False. The input shape to the model is 224 × 224 × 3, While the trainable parameters of the model are set to False. This is followed by the reshape layer, which comprises the Flatten layer and Dense layer with the SoftMax activation function to detect the driver’s drowsiness status. The parameters total 1,052,020, which is split into trainable and non-trainable parameters with 112,900 and 939,120, respectively. Figure 7 shows the details of MobileNet-V3 model architectures. Table 6 shows the details of MobileNet-V3 model architectures.

##### Hyperparameter Configuration for Training Deep Models

In this study’s experiment, the same training parameters for all driver drowsiness detection deep models during the ‘compile model’ phase to achieve the best results and to evaluate the performance between the deep learning models. The optimizer used is Adam optimizer with a default learning rate of 0.001, first-moment decay of 0.9, second-moment decay of 0.999, and epsilon was chosen as 10^−7^, and a True value was set for AMSGrad. AMSGrad is a stochastic optimization method that seeks to fix the convergence issue with Adam-based optimizers. The loss function chosen in the experiment is categorical cross-entropy given by the following Equation (1) as follows:

(1) $$Loss=-\sum_{c=1}^{M}y_{o,c}In\left(p_{o,c}\right)$$

where M is the number of classes; y is the binary indicator if c is the correct prediction for observation o; and p is the predicted probability for the observation being of class c. During the fit of deep learning models, each deep learning model completes approximately 200 epochs, with a batch size of 32. Table 7 illustrates the configuration of the training parameters for each model used in the experiment.

##### Deep Models Evaluation Procedure

To evaluate the performance of the deep learning models in the driver drowsiness detection case, the study uses various metrics: accuracy (ACC), loss, precision, recall, and F1-score. A confusion matrix is usually provided for each model to describe the performance, as illustrated in Table 8. A confusion matrix is a table used to evaluate the accuracy of a deep learning model for each class of dataset using a test dataset. The related terminology for a confusion matrix is described below:

- Accuracy is measured by the number of correct samples over the total number of samples. As given in Equation (2), Tp and Tn are the true positive and negative parameters, respectively, while Fp and Fn are the false positive and false negative values:
  (2) $$Accuracy\left(ACC\right)=\frac{Tp+Tn}{Tp+Tn+Fp+Fn}$$
- Precision also called the positive predictive value, indicates that the quantity means measuring the prediction of positive identifications is correct. As in Equation (3), precision presents the total number of samples as predicted as positive over the number of samples actually and predicted as positive:
  (3) $$Precision=\frac{Tp}{Tp+Fp}$$
- Recall or Sensitivity, also known as the positive rate, is the number of samples actually and predicted as positive from the total number of actually positive samples, as calculated in Equation (4):
  (4) $$Recall(Sensitivity)=\frac{Tp}{Tp+Fn}$$
- F1-score is useful in the evaluate the model on both precision and recall, as it considers the weighted average of recall and precision, as shown in Equation (5):
  (5) $$F1-score=\frac{2\times Precision\times Recall}{Precision+Recall}$$

#### 5.2.3. Phase 3: Optimization and Conversion Models

In the optimization and conversion models phase, quantization methods are employed to obtain a smaller model size. In the study’s experiment, two quantization methods, namely, quantization-aware training (QAT) and post-training quantization (PTQ), are implemented, as explained in Chapter 4. In QAT, the TensorFlow Model Optimization Toolkit is first installed, with this being a suite of tools for optimizing machine learning and deep learning models for deployment and execution [64]. The model is then applied to make the whole model aware of quantization: in other words, the study applies quantization to the whole model, that is, to the SqueezeNet, AlexNet, CNN, and MobileNet-V2 models. For the MobileNet-V3 model, the study conducts custom quantization by quantizing only the Dense layer. The setting for the compiled model is the hypermeter configuration setting. The models are trained using a training dataset and evaluated using a testing dataset. The value True is set to shuffle data, while the epochs for all models number 30. 

In the post-training quantization (PTQ) method, the two methods used are dynamic range quantization (DRQ) and full integer quantization (FIQ). In DRQ, optimization is adjusted as the default to quantize the weights of the models with 8-bit integers after completing the training. The study then converts the models using the TFlite Converter tools to TFLite format (serial format is based on the FlatBuffers library). It should be noted that before converting the models, the dataset is converted to 32-bit floating points. On the other hand, FIQ uses a representative dataset to convert the overall deep learning parameters, including weights and activation, to 8-bit integers.

#### 5.2.4. Phase 4: Running Interpreter

The TensorFlow Lite (TFLite) Interpreter is used to evaluate the quantized model, which is loaded and uses a testing dataset to evaluate the models in terms of accuracy. The quantized model is first assigned to the TFLite interpreter; then, the shape is adjusted to the type of Interpreter. The current study’s experiment resizes the input and output shapes based on the input and output test dataset shapes. The type of Interpreter is based on the input quantized model type, which, for DRQ, is “class ‘numpy.float32’” and for FIQ is “class ‘numpy.int8’”. The testing dataset is used as input to the Interpreter and is passed to ‘Invoke Interpreter’ to execute processing.

Notably, in QAT, the set tensor assigns the quantization value of the input Interpreter to Scale and the Zero Point variable; after that, the Interpreter divides the test images by their summation. The Interpreter outputs are passed to the Argmax function; their accuracy is then evaluated using metrics.

#### 5.2.5. Models’ conversion to C Array

In this phase, the TFLite microcontroller is used to generate a C byte array for the TFLite models utilizing standard tools to store it in a read-only program memory on the device [65]. The model format will be ‘model.cc’. Installing the XXD package creates the hex dump form of the given file, which can come back to the hex dump in the binary form [66]. By using XXD, the Unix command generates a C source file that contains the TFLite models as a char array. The TFLite models are converted into hex dump form with all parameters stored, and the architecture is expressed as a series of function calls that pass activations from one layer to the next. The output is generated “unsigned char g_model” which is in a single large file with a handful of entry points. Once the file is generated, it can be directly included in a program in the interactive development environment (IDE) or toolchain and compiled [9]. Notably, it is important to change the array declaration to ‘const’ for better memory efficiency on embedded platforms [65].

## 6. Results and Discussion

This section provides a detailed presentation of the results of the study’s experiment using a variety of metrics for each deep learning model in the training and evaluation phase. The section then presents the results of the size of each deep learning model after compression in the optimizations phase using post-training quantization (PTQ) (i.e., dynamic range quantization (DRQ), full integer quantization (FIQ), and aware quantization training (QAT)). In addition, a variety of metrics are used in the evaluation of the deep learning models. This Section is structured as follows: Section 6.1 presents the results of the training and evaluation of the models, while Section 6.2 presents the results of the optimization and conversion model phase. Section 6.3 describes the Interpreter phase results.

### 6.1. Training and Evaluation Model Results

#### 6.1.1. SqueezeNet Deep Model Result

**Figure 8:**
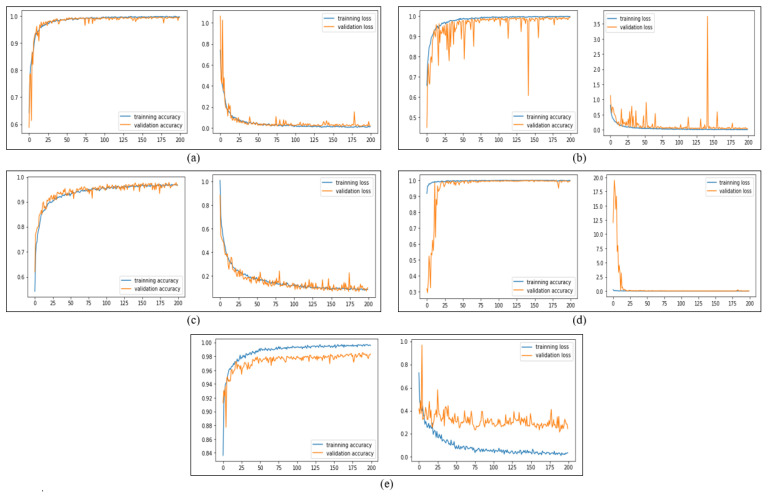
The accuracy and Loss values for evaluation DL models (**a**) SqueezeNet model (**b**) AlexNet model (**c**) CNN model (**d**) MobileNet-V2 model (**e**) MobileNet-V3 model.

The performance measures of the SqueezeNet model in the training and evaluation phase are based on accuracy and loss value, which are illustrated in Table 6. The results with augmentation and without augmentation are described for all three pre-processing methods SSD, Dlip, and Haar. The performance results showed the SSD method with augmentation achieved the best result in the SqueezeNet model with higher accuracy in the evaluation phase. The SSD method with augmentation obtained 0.9968 and 0.9947 accuracy for the training and evaluation phase, respectively. The loss value was 0.0107 for the training phase and 0.0205 for the evaluation phase. Figure 8a presents the results of the accuracy and loss value of the SqueezeNet model in the training and evaluation phase with the number of epochs.

**Figure 9:**
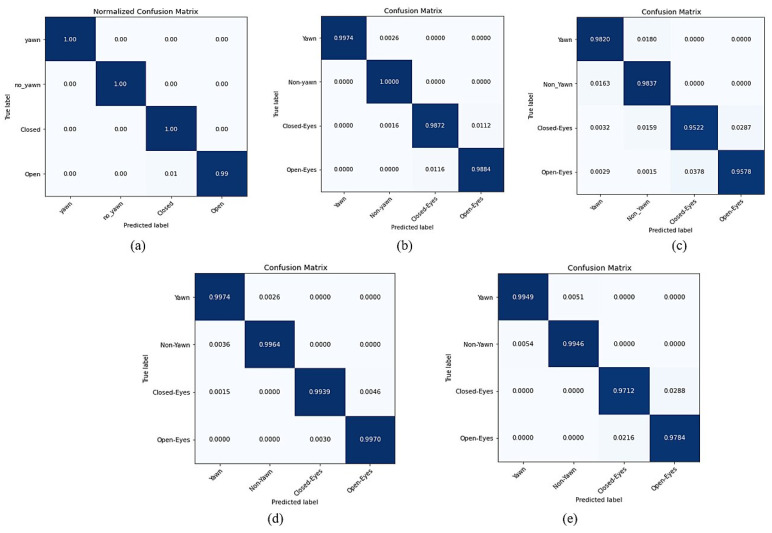
Confusion Matrix for DL mdoels (**a**) SqueezeNet model (**b**) AlexNet model (**c**) CNN model (**d**) MobileNet-V2 model (**e**) MobileNet-V3 model.

Based on this result, the performance results are next described in detail as precision, recall, and F1-score. In addition, for the confusion matrix. Table 6 presents the results of evaluating the SqueezeNet model using precision, recall, and F1-score. The results of the confusion matrix, even though they depend on the driver status of yawn or non-yawn, were both 1.00. The results of the confusion matrix for driver drowsiness based on eyes being closed or open were 1.00 and 0.99, respectively, as shown in Figure 9a.

#### 6.1.2. AlexNet Model Results

The modified AlexNet deep model in the driver drowsiness detection case achieved good performance for both types of driver status. Table 6 presents the results of training and evaluating the AlexNet model without augmentation and with augmentation for all the pre-processing methods. The results of the AlexNet model using the SSD method with augmentation outperformed the other methods in evaluating phase, achieving 0.9981 accuracies in the training phase and 0.9911 accuracies in the evaluating phase. However, the loss value in the training phase was 0.0071, and in the evaluating phase was 0.0341. Figure 8b shows the training and evaluation accuracy and loss of the AlexNet deep model.

Thus, based on this result, the study presented other measures, such as precision, recall, F1-score, and confusion matrix. The yawn and open eyes driver status outperformed precision as a driver status. Furthermore, the yawn driver status outperformed the recall metrics, while the non-yawn status achieved the best in F1-score, as in Table 6. Figure 9b presents the results of the confusion matrix for all types of driver drowsiness status.

#### 6.1.3. CNN Deep Model Results

The lightweight CNN model achieved high-performance results, with its low architecture complexity for detecting driver status. Table 6 shows the results of the CNN model using all pre-processing methods. The CNN model with augmentation using the SSD method achieved the best performance result of accuracy in the evaluation phase with 0.9734 and with a loss value of 0.0852. At the same time, it achieved 0.9637 accuracy in the training phase with a loss value of 0.1021. Figure 8c shows the performance measure for training and evaluation accuracy and loss value of the CNN deep model.

Building upon the results of the CNN model using the SSD method with augmentation as found to obtain the best performance result. The study introduced other metrics with results for precision, recall, and F1-score, as shown in Table 6. The study also introduced the confusion matrix, as shown in Figure 9c. The results of the confusion matrix for driver drowsiness based on the status of yawn and non-yawn indicate high performance with 0.9923 and 0.9909, respectively, while the results for closed eyes status was 0.9856 and for open eyes status was 0.937.

#### 6.1.4. MobileNet-V2 Deep Model Results

The results of the pre-trained MobileNet-V2 deep model in the driver drowsiness case achieved good performance in both types of driver status. Table 6 presents the results of training and evaluating the MobileNet-V2 model without augmentation and with augmentation for all three pre-processing methods, namely, the SSD, Dlip, and Haar. The results of the MobileNet-V2 model using the SSD method with augmentation outperformed the other methods in the evaluating phase, achieving 0.9998 accuracies in the training phase and 0.9960 accuracies in the evaluating phase. However, the loss value in the training phase was 0.0020, and in the evaluating phase was 0.0084. Figure 8d shows the training and evaluation accuracy and loss value of the MobileNet-V2 deep model with the number of epochs.

With these results in Table 9, the study presents other metrics for the SSD method with augmentation, such as precision, recall, and F1-score. The confusion matrix results of the MobilNet-V2 model based on the yawn status was 0.9974 and, for non-yawn status, was 0.9964, as shown in Figure 9d. Moreover, the confusion matrix of MobileNet-V2 achieved good results for detecting closed eyes status and open eyes status with 0.9939.

#### 6.1.5. MobileNet-V3 deep Model Results

The result for the pre-trained small MobileNet-V3 deep model was good in the training and evaluation phase for all methods of driver drowsiness detection. The SSD method with augmentation achieved high accuracy in the evaluation phase, with this followed by the Dlip method with augmentation and then the SSD method without augmentation. The SSD method with augmentation achieved 0.9956 accuracies in the training phase and 0.9832 accuracies in the evaluation phase, as depicted in Figure 8e. The loss values were 0.0352 and 0.2459 for the training and evaluation phases, respectively, as shown in Table 6.

Building upon the result in the previous table, the current study explained other results for precision, recall, F1 score, and the confusion matrix for the SSD method with augmentation. Table 6 shows the results for precision, recall, and F1-Score. Furthermore, Figure 9e shows the result of the confusion matrix. The result of the yawn status was 0.9949, followed by the non-yawn status with 0.9946. Moreover, the results of driver status of eyes closed or eyes open were 0.9712 and 0.9784, respectively.

### 6.2. Optimization and Conversion Models Results

The optimizing and conversion models phase aimed to compress and convert 32-bit floating-point deep learning models, training them to become 8-bit integer deep learning models using quantization methods. The models were then converted to TFLite format. The following subsections present the sizes of the original models (i.e., the trained models) as well as the size of the models after performing quantization and conversion to become the TFLite model.

#### 6.2.1. SqueezeNet Model Size Results

The SqueezeNet deep model showed superior size results, with the measured size of the original SqueezeNet model being 2.042 MB [59]. In the optimization and conversion model phase, DRQ obtained the best size measure for the SqueezeNet model at 0.141 MB, approximately equal to 144.384 KB. This meant that DRQ decreased the original model’s size by approximately 93.1%. This was followed by FIQ with 0.143 MB in size and QAT with 0.145 MB in size, as shown in Table 7.

#### 6.2.2. AlexNet Model Size Results

Moreover, after the current study modified the AlexNet deep model architecture, the model showed a high rate of reduction in size in the optimization and conversion phase compared to the size of the original model. The reduction results for all quantization methods were convergent. Table 7 shows the results of the DRQ method reducing the size of the original deep model by 86.4% (from 9.222 MB to 0.58 MB). This was followed by FIQ and QAT, which reduced the original model size by approximately 93.8% to 0.583 MB and 0.584 MB, respectively. Thus, the AlexNet deep model could reduce its memory footprint by at least 93.8%. Furthermore, without any accuracy reduction, the TFLite model achieved 0.9920 accuracies, the same accuracy performance compared to the original model without any quantization, around a 0.987 accuracy.

#### 6.2.3. CNN Model Size Results

The CNN deep model showed promising results in the reduction of deep learning model size in the optimization phase. The CNN model size results outperformed other deep learning models, with it acquiring the smallest model size. The CNN model’s size was a few megabytes, with the DRQ method achieving a 0.05 MB reduction to around 51 KB. This method reduced the original model size by 93.5% from 0.717 MB to 0.05 MB. Similarly, the results for QAT and FIQ were convergent with the DRQ results at 0.057 MB and 0.051 MB, respectively. The TFLite CNN deep model size consumed a few kilobytes of memory with a high level of accuracy. Thus, it has great potential to run smoothly on resource-constrained IoT devices.

#### 6.2.4. MobileNet-V2 Model Size Results

Table 10 shows the results of the pre-trained MobileNet-V2 deep model in the optimization and conversion phase. The DRQ method was found to be the best method for size reduction of the MobileNet-V2 model, reducing its size from 6.443 MB to 1.55 MB, in other words, by approximately 75.95%. Subsequently, the FIQ and the QAT methods achieved 1.703 MB and 1.705 MB in size, respectively.

#### 6.2.5. MobileNet-V3 Model Size Results

The size of the pre-trained MobileNet-V3 deep model, at 5.955 MB, was smaller than that of the original model. After quantization and conversion, the MobileNet-V3 achieved good size reduction using both DRQ and FIQ methods, resulting in 1.165 MB and 1.269 MB, respectively, which reduced the original size by approximately 75.95%. Using the QAT method, the size of the MobileNet-V3 deep model was 3.99 MB.

The results in Table 5 show that the DRQ obtained the best result in the compression of the model to reduce the size for all the models. It reduces the models four times more than the pre-trained models. The best result was in the CNN model with 0.05 MB. Followed by the FIQ method that shows the results of the reduction nearest to the DRQ. Whilst the QAT method achieved the lowest value in the size reduction of the models. Notably, in the Mobilenet-V3 model, the QAT does not achieve high reduction due to the quantization of the last layer.

### 6.3. Interpreter Phase Results

In the converting and optimization phase, the current study optimizes and converts the model to the TFLite format. After that, the interpreter was used to evaluate the performance accuracy of the TFLite models after using the quantization. Notice, in the QAT, the deep learning models were first trained and evaluated, then converted to the TFLite format, and used the interpreter to evaluate the deep learning models.

#### 6.3.1. SqueezeNet Deep Model Results

Table 11 presents the performance accuracy of the SqueezNet model after being converted to TFLite format by all quantization methods. In training and evaluation of the QAT method, the study undertook quantization of all layers of the models. As a result, the total parameters after quantization of the models were 120,302, divided into 118,420 trainable and 1,882 non-trainable. Using the SSD method with augmentation achieved the best accuracy result, with 0.9964 and 0.9889 accuracies in the training and evaluation phases, respectively. The loss value in the training phase was 0.0117, and in the evaluation phase was 0.0276. In evaluating the TFLite model phase using the Interpreter, the SSD method with augmentation obtained higher accuracy in DRQ, FIQ, and QAT with 0.99512, 0.9946, and 0.9889, respectively. This caused a drop in accuracy of less than 1% in comparison with the accuracy of the original model, which achieved a 0.9968 accuracy for the Dlip techniques with augmentation. Furthermore, the Haar technique without augmentation obtained a lower inference time. 

#### 6.3.2. AlexNet Deep Model Results

Table 12 presents the performance accuracy of the AlexNet deep model after conversion to TFLite format for all quantization methods. The results for the TFLite AlexNet model using the SSD method with augmentation outperformed the other quantization methods. The total parameters after conversion to the TFLite model or quantization were 599,461, divided into 598,532 trainable parameters and 929 non-trainable parameters. In addition, the QAT method achieved 0.9827 accuracy and 0.0690 value loss. In the Interpreter phase, the FIQ and DRQ achieved the best accuracy result with 0.9929 and 0.9924, respectively. These results indicated that no accuracy reduction occurred compared to the accuracy of the original model in the evaluation phase at 0.9925. Moreover, the Interpreter in the QAT phase obtained the lowest accuracy at 0.9827. 

#### 6.3.3. CNN Deep Model Results

Table 13 presents the performance accuracy of the CNN deep model after conversion to the TFLite format for all quantization methods. The total parameters of the TFLite in QAT numbered 46,677, divided into 46,574 trainable and 103 non-trainable parameters. The best performance results were achieved using the SSD method with augmentation in the QAT evaluation phase, with a 0.9616 accuracy achieved in the training phase and a 0.9654 accuracy achieved in the evaluation phase. The loss values were 0.1250 and 0.2303 for the training and evaluation phase, respectively. In the Interpreter phase, the accuracy results of the CNN model using the QAT method were similar. The results of quantization-aware training were 0.9689, FIQ 0.9645, and DRQ 0.9667. It achieved a less than 1% accuracy drop compared to the original model with 0.9667.

#### 6.3.4. MobileNet-V2 Deep Model Results

The pre-trained MobileNet-V2 model achieved good results in the model optimization and conversion phase. The TFLite MobileNet-V2 deep model achieved high results, as shown in Table 14. The results of the TFLite MobileNet-V2 model using the SSD method with augmentation outperformed the other quantization methods. With QAT, the model achieved a 0.9994 accuracy and a 0.9960 accuracy in the training and evaluation phase, respectively, with loss values of 0.0022 and 0.0144, respectively. In the Interpreter phase, QAT also achieved almost the best result with a 0.9960 accuracy, while DRQ achieved similar results with a 0.9964 accuracy. Inference times were 77.7016 seconds and 52.5048 seconds. The accuracy result, at 0.9960, was powerful compared to the accuracy of the original model. Furthermore, the FIQ method achieved a 0.9601 accuracy with an inference time of 81.8544 seconds, which caused an approximate 4% drop in accuracy from the original model, which achieved 0.9960.

#### 6.3.5. MobileNet-V3 Deep Model Results

The pre-trained MobileNet-V3 deep model obtained good performance in the optimization phase using quantization methods and after being converted to the TFLite model to detect the driver drowsiness statuses. After conducting QAT for the last layers, the total parameters numbered 1,052,026, with these divided into 112,900 trainable parameters and 939,126 non-trainable parameters. The number of parameters was less after quantization than the number of parameters of the original model. The whole quantized model did not support the MobileNet-V3 model, with the MobileNet-V3 architecture containing a TFOpLambda layer. Thus, only the last layer, the Dense layer, was quantized. The SSD method with augmentation obtained the best performance results, with a 0.9892 accuracy and a 0.9730 accuracy in the training and evaluation phase, respectively. The loss values were 0.1409 and 0.4253 in the training and evaluation phase, respectively. In the Interpreter phase, the results of the performance pre-trained MobileNet-V3 model using the SSD method with augmentation achieved the best performance results. The highest accuracy of 0.9729 was achieved by QAT, followed by DRQ with 0.9698. In contrast, FIQ achieved low accuracy of 0.6170, as shown in Table 15.

## 7. Discussion and Comparison

The main challenge of integrating deep learning models with IoT devices in driver drowsiness detection is the complexity of deep learning models, which require high computational costs. Thus, much training time is needed, as well as much consumption of the resources of IoT devices. The current study proposed five lightweight deep learning models to detect driver drowsiness more efficiently in terms of memory and complexity compared to deep learning models in prior studies. Three lightweight deep models were developed, namely, SqueezeNet, AlexNet, and CNN. In addition, the study adopted two pre-trained deep learning models, that is, MobileNet-V2 and MobileNet-V3, with their pre-training conducted on the ImageNet dataset. These models could detect driver drowsiness status of drowsy or non-drowsy from face images for both males and females, of different ages, with prescription eyeglasses and without glasses. Furthermore, the study used pre-processing methods, such as SSD, Dlip, and Haar, which were capable of detecting the faces of drivers in different situations, for instance, whether the driver was looking forward or to the side. In the driver’s drowsiness status, yawn means drowsy and non-yawn means non-drowsy. Additionally, these models could detect the status of driver’s eyes, with closed eyes and open eyes meaning that drivers were drowsy or non-drowsy. The various suggested models were evaluated in the training and evaluation phase based on their accuracy, recall, precision, and loss. In the Interpreter phase, accuracy was used to evaluate performance following conversion to TFLite models, with the size of the models also evaluated.

### 7.1. Training and Evaluation Phase

**Figure 10:**
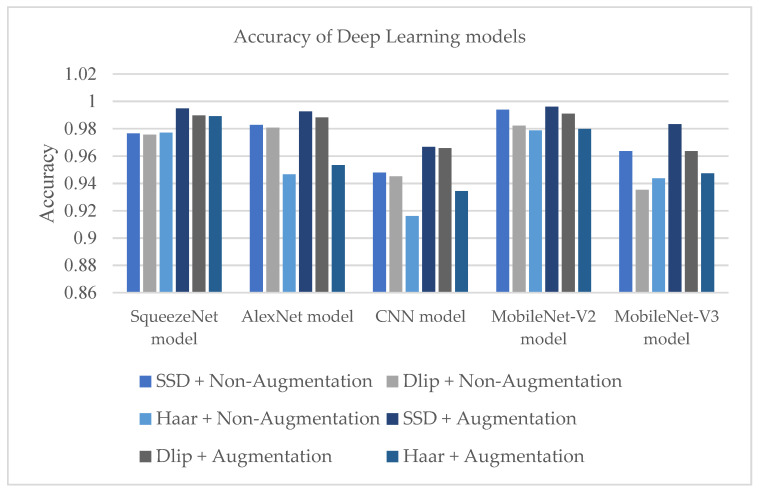
Accuracy for the five deep learning models with augmentation and non-augmentation datasets applied to the SSD, Dlip, and Haar methods.

The study evaluated the deep learning models in the evaluation phase based on their accuracy. Figure 10 shows the performance accuracy for all the deep learning models. The best results and the highest performance accuracy of the deep models were achieved by using the SSD method with augmentation in MobileNet-V2, SqueezeNet, and AlexNet models with a 0.9960 accuracy, a 0.9947 accuracy, and a 0.9911 accuracy, respectively. In addition, the SSD method without augmentation in the pre-trained MobileNet-V2 model showed a high level of accuracy with 0.9939. From the results, the study concluded that using the SSD method achieved the highest level of accuracy, with this method having the ability to accurately detect drivers’ faces from images while drivers were looking forward or to the side. After that, the study progressed to deep learning models to predict driver status. However, the CNN model had the lowest performance accuracy using the Haar method with a 0.9160 accuracy, although using the SSD method with augmentation achieved a 0.9658 accuracy. 

On the other hand, the MobileNet-V2, SqueezeNet, and AlexNet models using the SSD method with augmentation obtained the lowest loss values with 0.0084, 0.0205, and 0.0331. Conversely, the pre-trained MobileNet-V3 model obtained the highest loss value of 1.2577 using the Dlip method without augmentation and a loss value of 1.1663 using the Haar method with augmentation.

### 7.2. Optimization and Conversion Model Phase

**Figure 11:**
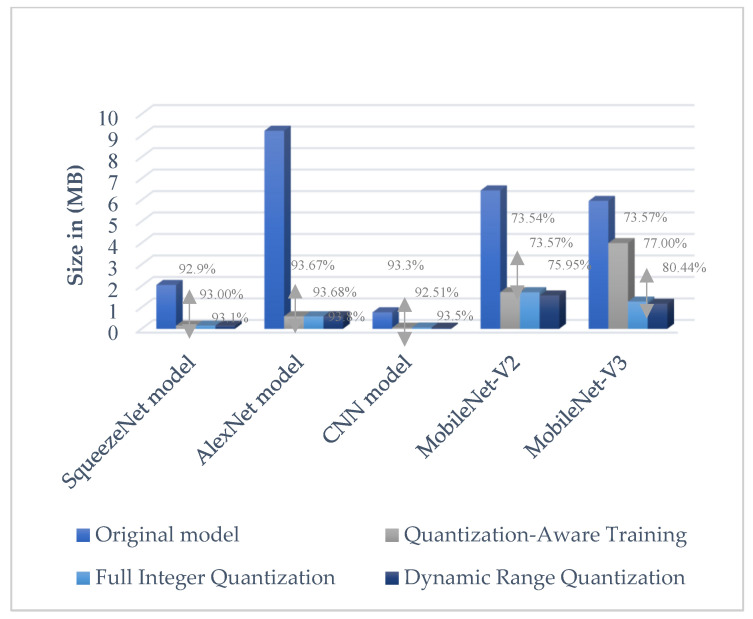
The size of the five deep learning models after optimization and conversion.

From this study’s experiment, the low variance was observable in the results that showed high performance when comparing the accuracy with model size. As shown in Figure 11, the quantization methods provided a significant decrease in model size with a small drop in accuracy. When implementation used fixed-point numbers on 8-bit integers, hence providing a reduction in the average power consumption, this also decreased inference time. As power consumption is a key parameter in IoT devices, shorter inference times are of interest as they make it possible to reduce the microcontroller’s operating frequency. In addition, execution using 8-bit integers also provides a significant reduction in memory footprint.

The current study’s results showed the CNN model using DRQ had the smallest model size with 0.05 MB, whereas no reduction occurred in the accuracy result when using this method. This was followed by the SqueezeNet model with an approximate size of 0.141 MB after quantization with a decrease in model size of 93.1%. Similarly, with quantization, a considerable decrease occurred in the AlexNet model size, which decreased from 9.222 MB to approximately 0.58 MB. The pre-trained models, namely, MobileNet-V2 and MobileNet-V3, had model sizes larger than our development model, with 1.550 MB and 1.165 MB, respectively. The MobileNet-V2 model, with 0.9960, showed higher performance accuracy than the MobileNet-V3 model, which achieved 0.9832. However, the MobileNet-V2 model obtained a high level of performance with a small drop in accuracy after the model was converted to the TFLite model. This was in contrast with the MobileNet-V3 model, which, after quantization and conversion, had a high level of variance and a drop in accuracy.

### 7.3. Interpreter Phase

Experiments are performed to check the performance of the deep learning models after using quantization methods. This study’s experiment achieved a high level of accuracy in the Interpreter phase in comparison to the accuracy of the original model without optimization. 

**Figure 12:**
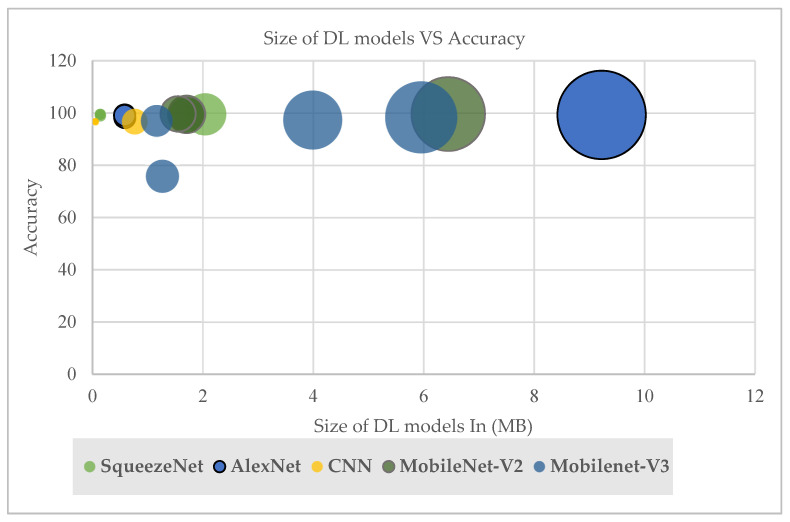
Size of the deep learning model in the evaluation phase and Interpreter phase for the five DL models.

In the QAT method, the accuracy of the evaluation phase was presented for all deep learning models. The pre-trained MobileNet-V2 deep model achieved the highest performance with a 0.9960 accuracy using the SSD method with augmentation, even though the current study conducts the whole quantization in the MobileNet-V2 model. The results showed a similar level of accuracy to the original models with no reduction in accuracy. At the same time, the SqueezeNet model achieved 0.9889 accuracy with the same method. The MobileNet-V2 model achieved a 0.9880 accuracy using the Dlip method with augmentation, while the AlexNet model achieved a 0.9840 accuracy using the SSD method with augmentation. Figure 12 illustrates the accuracy performance of QAT in the evaluation phase for all the models after 30 epochs.

In the Interpreter phase, the results clearly showed good performance with little drop in accuracy through using all the quantization methods, namely, QAT, FIQ, and DRQ. Firstly, in QAT, the MobileNet-V2 model achieved a high accuracy of 0.996 using the SSD method with augmentation. The mobileNet-V2 also showed a high-performance result of 0.9885 using the Dlip method with augmentation. Similarly, the SqueezeNet model achieved a 0.9884 accuracy using the SSD method with augmentation. This was followed by the AlexNet model with 0.9844 accuracy. Conversely, the MobileNet-V3 and CNN models achieved the lowest accuracy performance of the models, with a 0.9729 accuracy and a 0.9689 accuracy, respectively. Figure 12 shows the change in accuracy through QAT for all models in the Interpreter phase.

Secondly, the outcomes of the FIQ method results also showed that performance did not lead to a drop in accuracy, with the FIQ method quantizing the parameters to 8-bit integers of weights and activation functions. The best results of the deep learning models using the FIQ method were obtained by SqueezeNet and AlexNet models with a 0.9946 accuracy and a 0.9929 accuracy using the SSD method. This was followed by the MobileNet-V2 model using the Dlip method with a 0.9920 accuracy. The results showed that the drop in accuracy was less than 1% in comparison with the accuracy in the original model. Conversely, the FIQ method did not provide a substantial improvement over the MobileNet-V3 model. However, it caused high variance with a drop in accuracy of 17%, whereas the highest level of accuracy of the MobileNet-V3 model was achieved by the Dlip method with a 0.7846 accuracy.

Lastly, with the DRQ method, the study’s models were observed to achieve better results compared to the QAT and FIQ methods. The DRQ method achieved the lowest drop in accuracy in comparison with the accuracy of the original models. As outlined in Figure 12, the MobileNet-V2, SqueezeNet, and AlexNet models achieved higher results with 0.9964, 0.9951, and 0.9924 accuracies, respectively. The study observed that this did not lead to a drop in accuracy of less than 1% in comparison with the original model’s accuracy. 

To conclude the results of the study’s experiment, Figure 12 presents the accuracy of all deep learning models in the evaluation phase and the Interpreter phase versus the size of the models. Thus, despite the significant reduction in the size of the models compared to the original models, only a slight drop in accuracy was noted in comparison with the accuracy of the original model.

### 7.4. Comparison between Our Models and the Previous Studies

Table 16 introduces the comparison of the accuracy of the driver drowsiness detection method for deep learning models from prior research. Performance in the evaluation phase is also compared with prior studies of driver drowsiness detection on YawDD and Closed Eyes in the Wild (CEW) datasets. The accuracy of prior studies’ experiments ranged from 74.9% to 97.47%. The result of the current study’s experiment outperformed the current state-of-the-art studies. The current study’s model using SqueezeNet, AlexNet, MobileNet-V2, and MobileNet-V3 models achieved performance levels of 99.47%, 99.25%, 99.60%, and 98.32%, respectively, which were higher levels of performance than the results of prior research. The exception was the CNN model, which achieved the lowest result with 96.67% accuracy.

In the optimization and conversion phase, the size of the model was compressed to a lightweight model to enable it to be deployed on IoT devices that had constrained resources. Based on prior studies of driver drowsiness detection reviewed, the developed deep learning models are heavy in size and range between 236 MB and 10 MB. Thus, they are not appropriate for deployment on devices that have limited resources. The results of the study’s experiment showed good performance and a high level of accuracy with a few megabytes of deep learning model size. In comparison with other TinyML studies, the study’s proposed model obtained the smallest model size among the deep learning models while achieving a high level of performance, as demonstrated in Table 17. The CNN model achieved the smallest size with 0.05 MB, approximately equal to 51.2 KB, using DRQ, in comparison with another study which achieved the smallest model size of 138 KB. The size of our proposed SqueezeNet model is 2.042 MB after modification. In another study in [60], the authors reduced by 50 times the parameters of the model, so it became 4.8 MB. In addition, another study achieved 3.84 MB after modifying the original model’s architecture. The current study’s experiment obtained the smallest model size after optimization at 0.141 MB, equal to 144.384 KB. In terms of comparison with MobileNet-V3 models, to the best of the author’s knowledge, no research has applied quantization to the MobileNet-v3 small model. In previous studies, for example, in [72], the current study found that the authors applied the QAT method to the MobileNet-V3 large model that reduced the size of the model from 17 MB to 5 MB. In addition, in the study in [73], the author sought to reduce the size of models using different bottlenecks for convolution, hyper-parameter tuning, change of activation function, and introducing more expansion filters. The result achieved was to reduce the model from 15.3 MB to 2.3 MB. The complete code of our models was uploaded to GitHub [74].

## 8. Conclusions and Future Work

The current study employed TinyML to overcome the challenges of integrating deep learning models with IoT devices in smart cities. In this study, TinyML was applied to a driver drowsiness detection case study. To the best of the author’s knowledge, no prior research related to driver drowsiness detection studies has applied TinyML, as stated in the author’s recent co-authored published paper “TinyML: Enabling of Inference Deep Learning Models on Ultra-Low-Power IoT Edge Devices for AI Applications” [23]. In the current study, five lightweight deep learning models were evaluated to enable their integration with IoT devices that had low power and restricted resources. Three deep learning models were developed, namely, SqueezeNet, AlexNet, and CNN, while the study adopted two pre-trained models, namely, MobileNet-V2 and MobileNet-V3, to detect driver status.

Our five developed deep learning models achieved high accuracy results in state-of-the-art research related to driver drowsiness detection. MobileNet-V2, SqueezeNet, AlexNet, and MobileNet-V3 deep models achieved 0.9960, 0.9947, 0.9911, and 0.9832 accuracies, respectively, in identifying driver drowsiness status (i.e., yawning, non-yawning, closed eyes, and open eyes). The SSD pre-processing method outperforms the other methods in the performance of our deep learning models. For the size of the deep learning models, the CNN deep model outperformed, in comparison with other TinyML-related research, by achieving a model size of 0.05 MB. Furthermore, the modified SqueezeNet architecture achieved a smaller size of 0.141 MB compared to the existing SqueezeNet model. This was followed by the modified AlexNet model, which attained 0.58 MB, while the pre-trained MobileNet-V2 and MobileNet-V3 models achieved 1.55 MB and 1.165 MB, respectively. The results of the DRQ method caused an accuracy reduction of approximately less than 1%. The DRQ results outperformed other optimization methods, followed by QAT and FIQ methods.

These results indicate that without any accuracy degradation after performing optimization method with less than 1% approximately. Our experiment results point out; it has the great potential to run smoothly on resource-constrained IoT devices as the microcontroller. For instance, on the OpenMV H7 board, STM32H743VI, and STM32 Nucleo-144 H743ZI2. In addition, the SqueezeNet, AlexNet, and CNN models can be deployed on the SparkEdge development board Apollo3 Blue which has 1 MB of Flash memory and 384 KB of RAM. In addition, the CNN can also be deployed on an Arduino Nano Ple33 board that has flash memory with 256 KB and RAM of 32 KB.

Ongoing research aims to implement the current study’s deep learning models on microcontrollers as the family series of STM32 devices using CubeAI software or to implement them on Arduino Nano 33 BLE devices using Arduino software with testing in the real world.

## Figures and Tables

**Table 1:** Distributed images per driver status.

Categories of Driver Status	Number of Images	
	Raw Dataset	Augmented Dataset
Yawning	726	1377
Non-yawn	726	1940
Closed Eyes	1192	2132
Open Eyes	1231	2185
Total Dataset	3875 images	7634 images

**Table 2:** SqueezeNet deep model architecture.

Layer Type	Output Shape	Parameters
input_1 (InputLayer)	(None, 192, 192, 3)	0
conv2d (Conv2D)	(None, 192, 192, 32)	896
batch_normalization (BatchNormalization)	(None, 192, 192, 32)	128
Activation (Activation)	(None, 192, 192, 32)	0
conv2d_1 (Conv2D)	(None, 192, 192, 24)	792
batch_normalization_1(BatchNormalization)	(None, 192, 192, 24)	96
Activation (Activation)	(None, 192, 192, 24)	0
conv2d_2 (Conv2D)	(None, 192, 192, 24)	600
conv2d_3 (Conv2D)	(None, 192, 192, 24)	5208
concatenate (Concatenate)	(None, 192, 192, 48)	0
max_pooling2d (MaxPooling2D)	(None, 96, 96, 48)	0
conv2d_4 (Conv2D)	(None, 96, 96, 48)	2352
batch_normalization_2 (BatchNormalization)	(None, 96, 96, 48)	192
Activation_2 (Activation)	(None, 96, 96, 48)	0
conv2d_5 (Conv2D)	(None, 96, 96, 48)	2352
conv2d_6 (Conv2D)	(None, 96, 96, 48)	20,784
concatenate_1 (Concatenate)	(None, 96, 96, 96)	0
max_pooling2d_1 (MaxPooling2D)	(None, 48, 48, 96)	0
conv2d_7 (Conv2D)	(None, 48, 48, 64)	6208
batch_normalization_3 (BatchNormalization)	(None, 48, 48, 64)	256
Activation_3 (Activation)	(None, 48, 48, 64)	0
conv2d_8 (Conv2D)	(None, 48, 48, 64)	4160
conv2d_9 (Conv2D)	(None, 48, 48, 64)	36,928
concatenate_2 (Concatenate)	(None, 48, 48, 128)	0
max_pooling2d_2 (MaxPooling2D)	(None, 24, 24, 128)	0
conv2d_10 (Conv2D)	(None, 24, 24, 48)	6192
batch_normalization_4 (BatchNormalization)	(None, 24, 24, 48)	192
Activation_4(Activation)	(None, 24, 24, 48)	0
conv2d_11 (Conv2D)	(None, 24, 24, 48)	2352
conv2d_12 (Conv2D)	(None, 24, 24, 48)	20,784
concatenate_3 (Concatenate)	(None, 24, 24, 96)	0
max_pooling2d_3 (MaxPooling2D)	(None, 12, 12, 96)	0
conv2d_13 (Conv2D)	(None, 12, 12, 24)	2328
batch_normalization_5 (BatchNormalization)	(None, 12, 12, 24)	96
Activation_5(Activation)	(None, 12, 12, 24)	0
conv2d_14 (Conv2D)	(None, 12, 12, 24)	600
conv2d_15 (Conv2D)	(None, 12, 12, 24)	5208
concatenate_4 (Concatenate)	(None, 12, 12, 48)	0
global_average_pooling2d (GlobalAveragePooling2D)	(None, 48)	0
dense (Dense)	(None, 4)	196
Total params:	118,900	
Trainable params:	118,420	
Non-trainable params:	480	

**Table 3:** AlexNet deep model architecture.

Layer Type	Output Shape	Parameters
conv2d (Conv2D)	(None, 46,46,32)	11,648
batch normalization (BatchNormalization)	(None, 46,46,32)	128
Activation(Activation)	(None, 46,46,32)	0
max_pooling2d(MaxPooling2D)	(None, 23, 23, 32)	0
conv2d_1 (Conv2D)	(None, 13,13,64)	247,872
batch_normalization_1 (BatchNormalization)	(None, 13,13,64)	256
Activation_1(Activation)	(None, 13,13,64)	0
max_pooling2d_1 (MaxPooling2D)	(None, 6, 6, 64)	0
conv2d_2 (Conv2D)	(None, 4, 4, 128)	73,856
batch_normalization_2(BatchNormalization)	(None, 4, 4, 128)	512
Activation_2(Activation)	(None, 4, 4, 128)	0
max_pooling2d_2 (MaxPooling2D)	(None, 2, 2, 128)	0
flatten (Flatten)	(None, 512)	0
dense (Dense)	(None, 512)	262,656
dropout (Dropout)	(None, 512)	0
dense_1 (Dense)	(None, 4)	2052
Total params:	598,980	
Trainable params:	598,532	
Non-trainable params:	448	

**Table 4:** The proposed CNN deep model architecture.

Layer Type	Output Shape	Parameters
conv2d (Conv2D)	(None, 143, 143, 10)	280
conv2d_1 (Conv2D)	(None, 141, 141, 10)	910
max_pooling2d_1 (MaxPooling 2D)	(None, 70, 70, 10)	0
conv2d_2 (Conv2D)	(None, 68, 68, 10)	910
conv2d_3 (Conv2D)	(None, 66, 66, 10)	910
max_pooling2d_1 (MaxPooling 2D)	(None, 12, 12, 32)	0
flatten (Flatten)	(None, 4608)	0
dense (Dense)	(None, 4)	43,564
Total parameters:	46,574	
Trainable parameters:	46,574	
Non-trainable parameters:	0	

**Table 5:** MobileNet-V2 deep model architecture.

Layer Type	Output Shape	Parameters
MobileNet-V2	(None, 7, 7, 1280)	1,382,064
global_average_pooling2d (Glob alAveragePooling2D)	(None, 1280)	0
dense (Dense)	(None, 4)	5124
Total parameters:	1,387,188	
Trainable parameters:	1,360,548	
Non-trainable parameters:	26,640	

**Table 6:** The proposed MobileNet-V3architecture.

Layer Type	Output Shape	Parameters
MobileNet-V3	(None, 7, 7, 576)	939,120
flatten (Flatten)	(None, 28224)	0
dense (Dense)	(None, 4)	112,900
Total parameters:	1,052,020	
Trainable parameters:	112,900	
Non-trainable parameters:	939,120	

**Table 7:** Training parameters used for all the models in the experiment.

Training Parameters	Value
Learning rate	0.001
First-moment decay	0.9
Second-moment decay	0.999
Epsilon	10^−7^
AMSGrad	True

**Table 8:** Explanation of confusion matrix predicted and actual label.

	**Predicted**				
**Actual**		**Yawn**	**Non-Yawn**	**Closed-Eyes**	**Open-Eyes**
	Yawn	Pnn	Ppn	Pcn	Pln
	Non-Yawn	Pnp	Ppp	Pcp	Plp
	Closed-eyes	Pnc	Ppc	Pcc	Plc
	Open-eyes	Pnl	Ppl	Pcl	Pll

Pnn: Yawn class was correctly classified as Yawn. Ppn: Yawn class was incorrectly classified as Non-yawn. Pcn: Yawn class was incorrectly classified as Closed-eyes. Pln: Yawn class was incorrectly classified as Open-eyes. Pnp: Non-yawn was incorrectly classified as yawn. Ppp: Non-yawn class was correctly classified as Non-yawn. Pcp: Non-yawn class was incorrectly classified as closed-eyes. Plp: Non-yawn class was incorrectly classified as Open-eyes. Pnc: Closed-eyes class was incorrectly classified as yawn. Ppc: Closed-eyes class was incorrectly classified as Non-yawn. Pcc: Closed-eyes class was correctly classified as Closed-eyes. Plc: Closed-eyes class was incorrectly classified as Open-eyes. Pnl: Open-eyes class was incorrectly classified as Yawn. Ppl: Open-eyes class was incorrectly classified as Non-yawn. Pcl: Open-eyes class was incorrectly classified as Closed-eyes. Pll: Open-eyes class was correctly classified as Open-Eyes.

**Table 9:** Results of evaluation deep models using (Precision, Recall, F1-score, Accuracy, and Loss).

Model	Driver Status	Precision	Recall	F1-score	Accuracy	Loss
SqueezeNet	Yawn	1.00	1.00	1.00	0.99	0.02
	Non-yawn	1.00	1.00	1.00		
	Closed-Eyes	0.98	1.00	0.99		
	Open-Eyes	1.00	0.99	0.99		
AlexNet	Yawn	1.00	1.00	1.00	0.99	0.03
	Non-yawn	1.00	1.00	1.00		
	Closed-Eyes	0.99	0.99	0.99		
	Open-Eyes	0.99	0.99	0.99		
CNN	Yawn	0.97	0.98	0.97	0.96	0.09
	Non-yawn	0.97	0.98	0.98		
	Closed-Eyes	0.96	0.95	0.96		
	Open-Eyes	0.97	0.96	0.97		
MobileNet-V2	Yawn	0.99	1.00	0.99	0.99	0.002
	Non-yawn	1.00	1.00	1.00		
	Closed-Eyes	1.00	0.99	1.00		
	Open-Eyes	1.00	1.00	1.00		
MobileNet-V3	Yawn	0.99	0.99	0.99	0.98	0.24
	Non-yawn	1.00	0.99	1.00		
	Closed-Eyes	0.98	0.97	0.97		
	Open-Eyes	0.97	0.98	0.98		

**Table 10:** Deep learning models size after optimization and conversion.

DL Models	Original Model (MB)	Quantization Aware-Training (MB)	FIQ (MB)	DRQ (MB)
SqueezeNet model	2.042	0.145	0.143	0.141
AlexNet model	9.222	0.584	0.583	0.58
CNN model	0.767	0.051	0.057	0.05
MobileNet-V2	6.443	1.705	1.703	1.55
MobileNet-V3	5.955	3.99	1.268	1.165

**Table 11:** Quantization results of SqueezeNet model. * (Accuracy = ACC).

SqueezeNet Model			without Augmentation			Augmentation		
			SSD	Dlip	Haar	SSD	Dlip	Haar
QAT	Training:	* ACC:	0.9911	0.9956	0.9867	0.9964	0.9966	0.9941
		Loss:	0.0234	0.0113	0.0365	0.0117	0.0127	0.0189
	Evaluation:	ACC:	0.9687	0.9813	0.9643	0.9889	0.9812	0.9828
		Loss:	0.1194	0.0777	0.1188	0.0276	0.0692	0.0459
	Interpreter:	ACC:	0.9686	0.9812	0.9677	0.9884	0.9817	0.9814
		Time:	43.1166	41.8467	32.7756	89.5386	52.0805	55.7986
FIQ	Interpreter:	ACC:	0.9756	0.9765	0.9769	0.9946	0.9903	0.9883
		Time:	44.3879	41.0812	34.1582	92.7907	55.3195	57.5239
DRQ	Interpreter:	ACC:	0.9765	0.9756	0.9769	0.9951	0.9891	0.9890
		Time:	49.5479	46.6627	38.3709	109.5799	65.8292	64.8329

**Table 12:** Quantization results of AlexNet deep model.

AlexNet			without Augmentation			Augmentation		
			SSD	Dlip	Haar	SSD	Dlip	Haar
QAT	Training:	ACC:	0.9877	0.9888	0.9836	0.9922	0.9956	0.9857
		Loss:	0.0367	0.0289	0.0548	0.0275	0.0128	0.0435
	Evaluation:	ACC:	0.9452	0.9752	0.9206	0.9827	0.9778	0.9498
		Loss:	0.1876	0.0905	0.2731	0.0690	0.0845	0.1900
	Interpreter:	ACC:	0.9452	0.9752	0.9206	0.9827	0.9777	0.9498
		Time:	4.4683	5.9103	2.4513	8.2545	8.2408	3.0432
FIQ	Interpreter:	ACC:	0.9826	0.9814	0.9450	0.9929	0.9865	0.9510
		Time:	5.6406	7.2426	3.0830	8.2709	10.0502	3.8130
DRQ	Interpreter:	ACC:	0.9826	0.9807	0.9465	0.9924	0.9886	0.9534
		Time:	4.4341	5.8775	2.4573	10.3781	8.2117	3.0762

**Table 13:** Quantization results of CNN deep model.

CNN			without Augmentation			Augmentation		
			SSD	Dlip	Haar	SSD	Dlip	Haar
QAT	Training:	ACC:	0.9638	0.9542	0.9548	0.9616	0.9668	0.9519
		Loss:	0.1013	0.1236	0.1250	0.0995	0.0922	0.1257
	Evaluation:	ACC:	0.9400	0.9342	0.9145	0.9690	0.9613	0.9427
		Loss:	0.2227	0.1600	0.2303	0.0788	0.0913	0.1767
	Interpreter:	ACC:	0.9408	0.9341	0.9145	0.9689	0.9618	0.9438
		Time:	4.0409	4.3978	3.9754	13.8674	8.6379	3.3356
FIQ	Interpreter:	ACC:	0.9469	0.9451	0.9129	0.9645	0.9652	0.9330
		Time:	5.174	4.6237	4.0452	13.4254	9.9634	3.8518
DRQ	Interpreter:	ACC:	0.9486	0.9451	0.9145	0.9667	0.9658	0.9354
		Time:	1.9051	1.9344	2.2832	6.4634	5.8682	1.5095

**Table 14:** Quantization results of MobileNet-V2 deep model.

MobileNet-v2			without Augmentation			Augmentation		
			SSD	Dlip	Haar	SSD	Dlip	Haar
QAT	Training:	ACC:	0.9963	0.9940	0.9928	0.9994	0.9968	0.9903
		Loss:	0.0088	0.0151	0.0184	0.0022	0.0051	0.0296
	Evaluation:	ACC:	0.9748	0.9751	0.9710	0.9960	0.9880	0.9176
		Loss:	0.1686	0.1262	0.2841	0.0144	0.0832	0.5409
	Interpreter:	ACC:	0.9747	0.9740	0.9709	0.9960	0.9885	0.9163
		Time:	45.4991	31.5628	21.0755	77.7016	56.8664	26.6903
FIQ	Interpreter:	ACC:	0.9939	0.9800	0.9709	0.9601	0.9920	0.9796
		Time:	45.0814	33.4733	21.8219	81.8544	59.8576	0.00013
DRQ	Interpreter:	ACC:	0.9939	0.9810	0.9786	0.9964	0.9914	0.9796
		Time:	30.9504	22.4492	14.7802	52.5048	40.5510	19.0597

**Table 15:** Quantization results of MobileNet-V3 deep model.

MobileNet-v3			without Augmentation			Augmentation		
			SSD	Dlip	Haar	SSD	Dlip	Haar
QAT	Training:	ACC:	0.9922	0.9136	0.9867	0.9892	0.9814	0.9877
		Loss:	0.0907	0.1620	0.1283	0.1409	0.2316	0.1249
	Evaluation:	ACC:	0.9618	0.9202	0.9402	0.9730	0.9658	0.9415
		Loss:	0.6607	1.4519	2.2577	0.4253	0.8601	1.7640
	Interpreter:	ACC:	0.9530	0.9202	0.9401	0.9729	0.9657	0.9414
		Time:	4.7089	4.3929	3.9644	9.0482	4.8462	2.2009
FIQ	Interpreter:	ACC:	0.6993	0.7846	0.5650	0.6170	0.7576	0.6224
		Time:	24.1468	21.5367	23.5531	45.0350	37.1020	17.1534
DRQ	Interpreter:	ACC:	0.9409	0.9212	0.9067	0.9698	0.9327	0.9235
		Time:	8.3107	8.2792	6.8613	15.6946	13.3162	6.1322

**Table 16:** Accuracy comparison with prior research in evaluation phase.

Reference	Datasets	DL Models	Performance
[59]	Wild (CEW) dataset	CNN	ACC: 95%
[36]	YawDD dataset	2s-STGCN	ACC: 93.4%
[67]	YawDD dataset	CNN	ACC:93.83%
[40]	Wild (CEW) dataset	CNN	ACC:95.65%
[68]	YawDD dataset	Kalman + TLD + CNN	ACC:92%
[69]	YawDD dataset	SDM algorithm + CNN	ACC:89.55%
[70]	Wild (CEW) dataset YawDD dataset	CNN	ACC:96%
[26]	YawDD	MobileNetV3	ACC:97.26%
			ACC:97.47%
[71]	YawDD	HyMobLSTM	ACC: 98.4%
Our proposed	YawDD and Wild (CEW) dataset	SqueezeNet	ACC: 99.47%
		AlexNet	ACC: 99.25%
		CNN	ACC: 96.67%
		MobileNet-V2	ACC: 99.60%
		MobileNet-V3	ACC: 98.32%

**Table 17:** Accuracy comparison with prior research in Interpreter phase.

Study	DL Models	Original Model (MB)	QAT (MB)	FIQ (MB)	DRQ (MB)
[75]	CNN	15	-	-	2.4
[76]	SqueezeNet	8	-	-	0.761
	Modified SqueezeNet	3.84			0.376
	proposed CNN	1.52			0.134
[77]	CNN	1	-	0.180	-
[78]	SqueezeNet model	6.1	0.8	-	0.8
	MobileNet-V2	-	2.6	-	-
[79]	MobileNet-V2	1.6	-	-	0.571
[73]	MobileNet-V2	-	-	-	1.95
Our proposed	SqueezeNet	2.042	0.145	0.143	0.141
	AlexNet model	9.222	0.584	0.583	0.58
	CNN model	0.767	0.051	0.057	0.05
	MobileNet-V2	6.443	1.705	1.703	1.55
	MobileNet-V3	5.955	3.99	1.268	1.165

## Data Availability

Publicly available models’ codes were used in this study. This code can be found here: https://github.com/nourahnasser15/TinyML (accessed on 10 May 2023).
